# Pharmacological Properties, Therapeutic Potential and Molecular Mechanisms of JWH133, a CB2 Receptor-Selective Agonist

**DOI:** 10.3389/fphar.2021.702675

**Published:** 2021-07-30

**Authors:** Hebaallah Mamdouh Hashiesh, Charu Sharma, Sameer N. Goyal, Niraj Kumar Jha, Shreesh Ojha

**Affiliations:** ^1^ Department of Pharmacology and Therapeutics, College of Medicine and Health Sciences, United Arab Emirates University, Al Ain, United Arab Emirates; ^2^ Department of Internal Medicine, College of Medicine and Health Sciences, United Arab Emirates University, Al Ain, United Arab Emirates; ^3^ Shri Vile Parle Kelavani Mandal’s Institute of Pharmacy, Dhule, India; ^4^ Department of Biotechnology, School of Engineering and Technology (SET), Sharda University, Greater Noida, India; ^5^ Zayed Bin Sultan Center for Health Sciences, United Arab Emirates University, Al Ain, United Arab Emirates

**Keywords:** cannabinoid receptor agonist, cannnabinoids, JWH133, synthetic cannabinoids, cannabinoid agonists

## Abstract

The endocannabinoid system has attracted attention as a pharmacological target for several pathological conditions. Cannabinoid (CB2)-selective agonists have been the focus of pharmacological studies because modulation of the CB2 receptor (CB2R) can be useful in the treatment of pain, inflammation, arthritis, addiction, and cancer among other possible therapeutic applications while circumventing CNS-related adverse effects. Increasing number of evidences from different independent preclinical studies have suggested new perspectives on the involvement of CB2R signaling in inflammation, infection and immunity, thus play important role in cancer, cardiovascular, renal, hepatic and metabolic diseases. JWH133 is a synthetic agonist with high CB2R selectivity and showed to exert CB2R mediated antioxidant, anti-inflammatory, anticancer, cardioprotective, hepatoprotective, gastroprotective, nephroprotective, and immunomodulatory activities. Cumulative evidences suggest that JWH133 protects against hepatic injury, renal injury, cardiotoxicity, fibrosis, rheumatoid arthritis, and cancer as well as against oxidative damage and inflammation, inhibits fibrosis and apoptosis, and acts as an immunosuppressant. This review provides a comprehensive overview of the polypharmacological properties and therapeutic potential of JWH133. This review also presents molecular mechanism and signaling pathways of JWH133 under various pathological conditions except neurological diseases. Based on the available data, this review proposes the possibilities of developing JWH133 as a promising therapeutic agent; however, further safety and toxicity studies in preclinical studies and clinical trials in humans are warranted.

## Introduction

The endocannabinoid system comprises cannabinoid receptors (CB1R and CB2R), which play pivotal roles in various human biological and pathological conditions. Substantial effort has been focused on developing ligands for CB1R and CB2R, leading to hundreds of phyto- and synthetic cannabinoids with variable affinities linked to the treatment of several disorders (An et al., 2020). The endocannabinoid signaling pathway restores homeostasis after damage; thus, it is the basis of therapeutic approaches to pain, inflammation, cancer, cardiovascular, and metabolic and neurodegenerative disorders (Fulmer and Thewke, 2018; Cristino et al., 2020). CB1R and CB2R also mediate the bioactivities of several phytocannabinoids (Morales et al., 2017), suggesting the importance of these receptors in the pharmacological functions of the cannabis plant. These findings encouraged the ongoing development of diverse synthetic cannabinoids with similar or different structures compared with endo- and phytocannabinoids.

The CB2R is a G protein-coupled receptor that regulates intracellular mechanisms by coupling with Gi/o proteins (Howlett, 2005). CB2R inhibits adenylyl cyclase activity to produce cyclic adenosine monophosphate (cAMP) and mediates mitogen-activated protein kinase (MAPK) activation (Bouaboula et al., 1996). As a therapeutic target, CB2R has significant advantages. First, CB1R is primarily localized in the human brain and is primarily responsible for the psycho-activity of D9-tetrahydrocannabinol (THC) and the harmful psychiatric adverse effects of CB1R ligands (Zou and Kumar, 2018). Conversely, CB2R is mainly expressed in the peripheral tissues, including the immune system, and regulates immunologic function, cell migration, and cytokine secretion (Jordan and Xi, 2019). CB2R is expressed to a lesser degree in the brain, although at lower levels than CB1R (Zou and Kumar, 2018). Despite the lower levels of CB2R expression in the peripheral and central nervous system, CB2R plays a key role in nociception and neuroinflammation (Morales et al., 2016). Researchers have developed selective CB2R agonists with remarkable *in vitro* and *in vivo* effectiveness and no undesired psychotropic effects. Examples of such CB2R selective agonists are JWH015, HU308, JWH133 and GW-405833 (Hanuš et al., 1999; Valenzano et al., 2005; Verty et al., 2015; Çakır et al., 2019b). Animal studies have shown that CB2R stimulation modulates several pathophysiological processes (Aghazadeh Tabrizi et al., 2016) and is implicated in controlling different pathological conditions, including pain (Shang and Tang, 2017), inflammation (Turcotte et al., 2016), atherosclerosis (Carbone et al., 2014), diabetes (Basha and Sankaranarayanan, 2014), cancer (Elbaz et al., 2017), and cardiovascular disease (Steffens and Pacher, 2012). A clinical study of a CB2R agonist demonstrated effective mitigation of neuropathic pain (Gertsch et al., 2008). The neuroprotective effects of JWH133 will be comprehensively reviewed in another review. Thus, CB2R-specific therapeutic targeting is promising for discovering new therapies without adverse psychoactive effects associated with CB1R.

### Synthetic Cannabinoids

Synthetic cannabinoids are diverse in chemical structure and function. They were initially used as pharmacological tools for delineating the cannabinoid receptor-induced activity (Howlett and Abood, 2017). Thus, their structural features allow them to bind to one of the recognized cannabinoid receptors found in human cells, CB1 and/or CB2 (Hervás, 2017). Some of these synthetics appeared on the market as substitutes to phytocannabinoids for recreational drug use. Diverse synthetic cannabinoids have been developed recently with subtle structural changes (Morales et al., 2016; Hervás, 2017). These synthetic cannabinoids are structurally classified as classical, non-classical, amino-alkyl indoles, and eicosanoids (Badal et al., 2017), and many have been used in pharmacological studies, including those on structure-activity relationships, receptor binding, and drug mechanisms of action.

New selective CB2 agonists are now the focus of academic and commercial efforts, and a growing number of preclinical and *in vitro* studies have yielded encouraging findings. However, there has been limited success in clinical trials owing to a lack of translation from animal models to humans and differences among species (Morales et al., 2016; Ghonim et al., 2019; Mugnaini et al., 2019). The most extensively used pharmacological agent is the classical CB2R-selective cannabinoid JWH133 produced by Dr John Huffman Huffman et al, (1999). JWH133 binds with greater affinity to CB2R than CB1R and acts as a potent CB2R-selective agonist (Huffman et al., 1999).

### JWH133

JWH133 is a synthetic agonist devoid of psychogenic activity, with 200-fold greater CB2R selectivity than CB1R, with Ki of 3.4 nM and inhibitor constant of 677 nM (Huffman et al., 1999). JWH133 had no CB1R activity, such as antinociceptive, cataleptic, and hypothermic activities, in mouse cannabinoid triads (Soethoudt et al. (2017)). JWH133 is a highly selective full agonist of mCB2R but functionally inactive on hCB1R, with a maximum activity of only 20% at 10 mM, without off-target activities at active concentrations. Moreover, it has a moderate volume of distribution (1–3 l kg^−1^), with a half-life of only 1 h.

JWH133 belongs to the class of Δ8-tetrahydrocannabinol derivatives, which resembles the Δ9-tetrahydrocannabinol. Particularly, the research team of Huffman et al. revealed that the deletion of the phenolic OH group from HU210, non-selective CBRs agonist (Mechoulam et al., 1990), to obtain JWH051, did not markedly affect affinity for CB1R, but significantly increased CB2R affinity and selectivity (Huffman et al., 1996). The additional removal of alcoholic group and further modifications of the alkyl chain resulted in more CB2R-selective ligands, among them, JWH133 is remarkable: it is a potent CB2R agonist, with a Ki of 3.4 nM and a high selectivity for CB2R (around 200 folds over CB1R) (Huffman et al., 1999; Pertwee, 1999). A Comparision of the binding type and affinity of JWH133 with main phytocannabinoids are summarized in Table 1. The most significant plant-derived cannabinoid is Δ9-tetrahydrocannabinol (Δ9-THC). The psychogenic effects of cannabis are mostly attributed to partial agonistic activity of Δ9-THC at CB1Rs (Turner et al., 2017; Amin and Ali, 2019). In addition, Δ9-THC is also featured as a partial agonist at CB2Rs (Pertwee, 2008; Turner et al., 2017). Moreover, it has been shown that cannabidiol (CBD) has a very low affnity for CB1R and CB2R (Turner et al., 2017). CBD acts as an antagonist/inverse agonist at certain concentrations below which it binds to both CB1 and CB2 orthosteric sites (Badal et al., 2017). Lately, various studies have displayed that CBD acts as a negative allosteric modulator of CB1R, which modifies the potency and effcacy of the orthosteric ligands but does not activate the receptor itself (Chung et al., 2019; Tham et al., 2019). For CB2R, CBD acts as a partial agonist (Tham et al., 2019). Comparing with another natural cannabinoid with high selectivity to CB2R, β-caryophyllene (BCP), which selectively and competitively interact with the CP55,940 binding site (i.e., THC binding site) of the CB2R, with 165-fold selectivity over CB1R, where it showed a weak partial agonism (Gertsch et al., 2008).

**TABLE 1:** A comparision of JWH133 and main phytocannabinoids in terms of binding type and binding affinity.

Cannabinoids	Binding type/CB	CB1 K_i_ value (nM)	CB2 K_i_ value (nM)	References
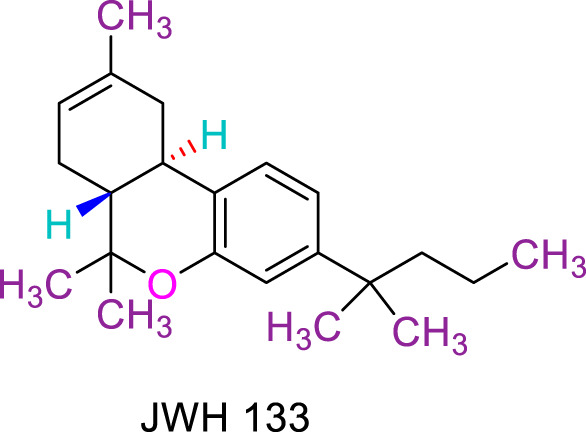	Full agonist/CB2	677	3.4	Pertwee et al. (2010)
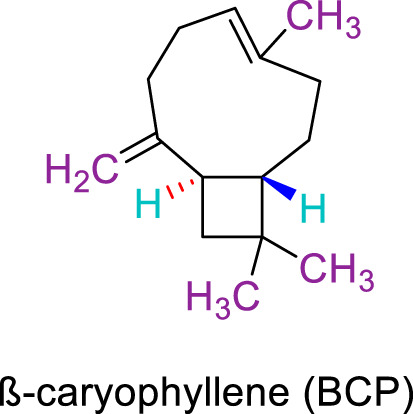	Full agonist/CB2	NA	155	Gertsch et al. (2008)
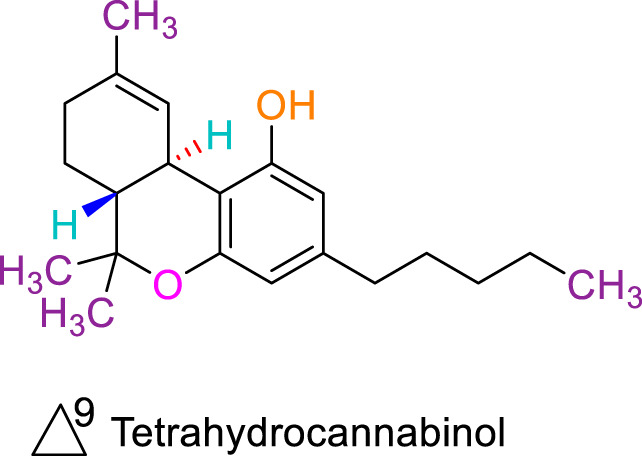	Partial agonist/CB1,CB2	5 to 80	1.7 to 75	Turner et al. (2017)
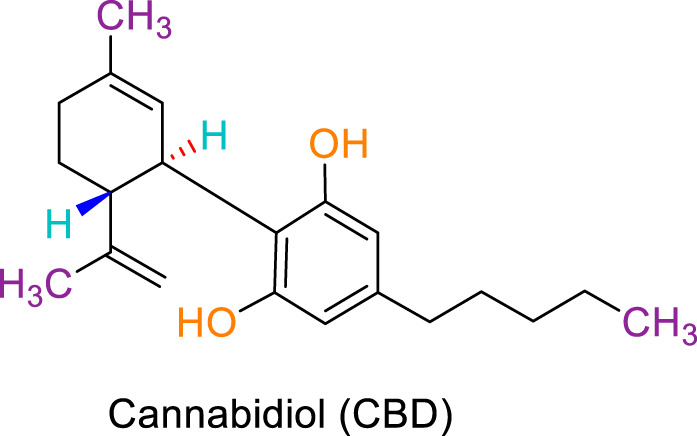	Antagonist/inverse agonist, negative allosteric modulator/CB1	73 to >10,000	370 to >10,000	Turner et al. (2017)
	Partial agonist/CB2			
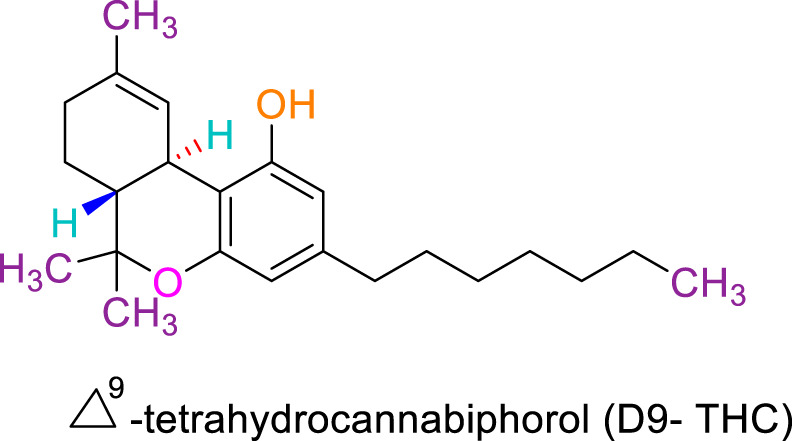	Agonist/CB1,CB2	1.2	6.2	Citti et al. (2019)

**FIGURE 1:**
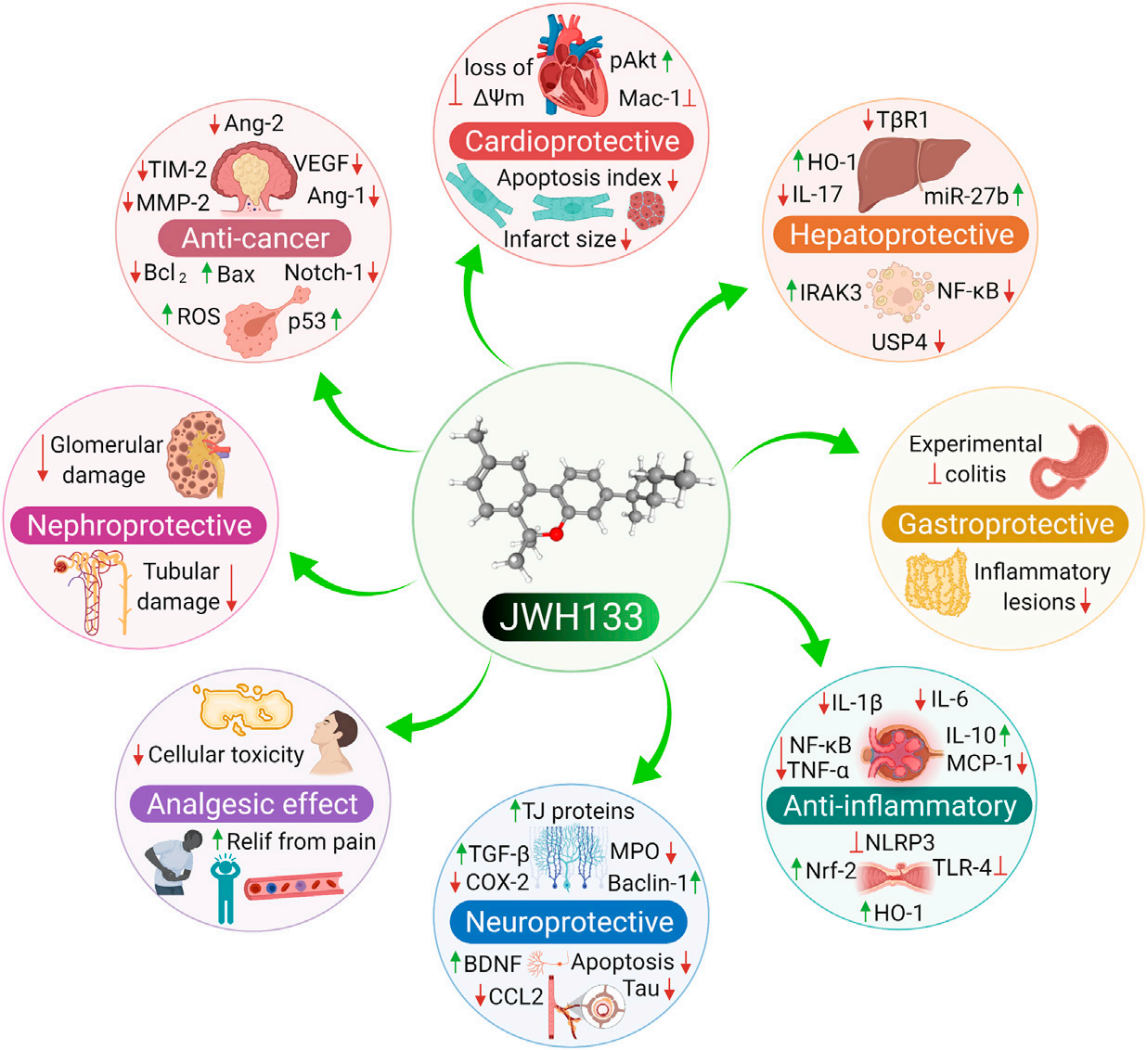
The Pharmacological properties of JWH133.

JWH133 exhibits anticancer (Sánchez et al., 2001; Qamri et al., 2009), cardioprotective (Yu et al., 2019), hepatoprotective (Wu et al., 2019), gastroprotective (Tartakover Matalon et al., 2020), nephroprotective (Feizi et al., 2008), anti-inflammatory (Çakır et al., 2020), antihyperalgesic (Cabañero et al., 2020), and immunomodulatory activities (Zhu et al., 2019). It has also been demonstrated to exert neuroprotective effects in Parkinson’s disease, ischemic stroke, depression, anxiety, Alzheimer’s disease, epilepsy, and neuropathic pain (Kruk-Slomka et al., 2015; Sheng et al., 2019; Cao et al., 2020; Ivy et al., 2020; Jia et al., 2020; Jing et al., 2020; Zhao et al., 2020). The neuroprotective role of JWH133 has been well demonstrated in a large number of experimental studies and currently not included in the present study due to space constraints. The neuroprotective effects of JWH133 will be reviewed comprehensively in another successive review. Several *in vitro* and animal studies have verified the biological properties of JWH133. The pharmacological properties of JWH133 are depicted in Figure 1. The pharmacological activities, mechanism and therapeutic potential of JWH133 in the *in vivo* studies and in the *in vitro* studies are summarized in Tables 2 and 3, respectively.

**TABLE 2:** Pharmacological activities, mechanism and therapeutic potential of JWH133 in the *in vivo* studies.

Experimental model	JWH133 doses	Indication/Disease	Demonstrated actions and mechanisms	References
Compound 48/80-induced inflammation in BALB/cJBom mice	20 and 20 µg/mouse i.p	Inflammation		Jonsson et al. (2006)
Cecal ligation and puncture (CLP)-induced polymicrobial sepsis model in Sprague-Dawley rats	0.0, 1, and 5 mg/kg, i.p	Inflammation	Inhibits the apoptosis and NF-κB signaling	Çakır et al. (2020)
C57Bl/6J mice injected with LPS-induced vascular inflammation	10 mg/kg ip	Atherosclerosis	Attenuates the TNF-α- and/or endotoxin induced expression of ICAM-1 and VCAM-1 and vascular endothelium adhesion	Rajesh et al. (2007)
Shear stress-induced atherogenesis and plaque vulnerability in apoE^−/−^ mice	5 mg/kg, i.p. for 5 days/week	Atherosclerosis	Suppresses neutrophil production of MMP-9 via attenuation of ERK1/2 phosphorylation	Montecucco et al. (2012)
Balloon-induced neointima in WT, ApoE^−/−^, CB2^−/−^ mice	5 mg/kg, i.p. 1 h before surgery and for 28 days after	Atherosclerosis	Modulates neointima formation via decreasing of proliferation, macrophage infiltration, and smooth muscle cell content	Molica et al. (2012)
Monosodium iodoacetate-induced osteoarthritis pain in Sprague-Dawley rats	1 mg/kg, s.c, for 28 days post-MIA injection	Osteoarthritis	Stimulation of CB2R diminished central sensitization process, leading to mitigation of pain behavior	Burston et al. (2013)
Subcutaneous xenografts mice and male PyMT transgenic mice	5 mg/kg, i.p. for 4 weeks	Breast cancer	Modulates COX-2/prostaglandin E2 signaling pathways	Qamri et al. (2009)
			⁃Induces cell cycle arrest and apoptosis	
MMTV-neu mice, a model of ErbB2-driven metastatic breast cancer	0.05 mg/animal/day, twice a week for 90 days	Breast cancer	Suppression of the pro-tumorigenic Akt pathway	Caffarel et al. (2010)
Rag-2^_/_^ mice, a mouse model of glioma	50 μg for 8 days, intratumoral	Brain cancer	Induces apoptosis via ceramide synthesis and ERK1/2 activation	Sánchez et al. (2001)
Glioma and astrocytoma xenografts	50 μg/d for 8 days or 25 days, intratumoral	Brain cancer	inhibition of vascular endothelial cell migration and survival as well as the decrease in expression of proangiogenic factors (VEGF and angiopoietin-2) and MMP-2 in the tumors	Blázquez et al. (2003)
Glioma xenografts mice	50 μg/d for 8 days, peritumorally	Brain cancer	Downregulates MMP-2 via inhibiting sphingolipid ceramide synthesis	Blázquez et al. (2008)
Nude mice inoculated with glioma cells	1.5 mg/kg, s.c	Brain cancer	Decreases efficiency of glioma stem cells and glioma formation due to reduced neurosphere formation and cell growth	Aguado et al. (2007)
SCID CB-17 mice inoculated with A549 cells	1 mg/kg, peritumorally for 28 days	Lung cancer	Decreases tumor proliferation and neo-vascularization along with enhanced apoptotic death	Preet et al. (2011)
Nude mice inoculated with PDV.C57 epidermal tumor cells	1,580 μg for 11 days locally infused at a rate of 0.52 μl/h	Skin cancer	Interferes with the tumor angiogenic switch together with the direct stimulation of apoptosis on tumor cells, which in turn inhibits tumor proliferation	Casanova et al. (2003)
			Abolishes EGFR function	
Nude mice bearing B16 melanoma cells	50 μg/day, daily for 8 days	Skin cancer	Rise in apoptosis and reduction of tumor vascularization, and vascular density	Blázquez et al. (2006)
Quetiapine-induced cardiotoxicity in Balb/C mice	5 mg/kg, i.p. for 21 days	Cardiotoxicity	Modulates necroptosis process	Li et al. (2019b)
Ethanol-induced cardiotoxicity in C57BL/6J mice	3 mg/kg, i.p. 1 h before ethanol administration for 30 or 45 days	Cardiotoxicity	Attenuates RIP1/RIP3/MLKL-mediated necroptosis	Liu et al. (2020b)
Clozapine-induced cardiotoxicity in C57BL/6J mice	2 mg/kg, i.p. before clozapine administration for 14 days	Cardiotoxicity	Attenuates myocardial inflammation, fibrosis, and myocardial injury	Li et al. (2019a)
I/R injury of the C57Bl/6 mouse heart	20 mg/kg, i.p. 5 min before reperfusion	Myocardial infarction	Inhibition of oxidative stress and neutrophil recruitment and activation of ERK 1/2 and STAT3 pathway	Montecucco et al. (2009)
I/R injury of the Sprague-Dawley rats heart	20 mg/kg, I.V. 5 min before ischemia	Myocardial infarction	Prevents apoptotic cell death via suppressing the intrinsic mitochondrial apoptotic process and implication of the PI3K/Akt signaling pathway	Li et al. (2013a)
I/R injury of the C57Bl/6 WT and CB2^−/−^ mice heart	3 mg/kg, I.V. 5 min before reperfusion	Myocardial infarction	Prevention of oxidative stress-induced cardiac myocyte and fibroblast apoptosis and the suppression of myofibroblast activation	Defer et al. (2009)
I/R injury of the C57Bl/6 mouse heart	1, 3, and 10 mg/kg, i.p. 5 min before ischemia	Myocardial infarction	Modulation of NLRP3 inflammasome pathway	Yu et al. (2019)
HFD-induced obese mice model (60% kcal fat content) for 10 weeks	5, 10 mg/kg, i.p. for 21 days	Obesity	Attenuates pro-inflammatory M1 macrophage cytokines through the Nrf2/HO-1 mechanism	Wu et al. (2020)
db/db mice	0.15, 0,5, 1 and 3 mg/kg, s.c	Diabetic neuropathy	⁃Activation of antioxidant Nrf2/HO-1pathway potentiated the antiallodynic effects	McDonnell et al. (2017)
Seven-day-old swiss CD-1 mice	1.5 mg/kg for 5 h (acute treatment) or for 5 consecutive days per week for 2 and 3 weeks (chronic treatment)	Spermatogenesis	Accelerates the spermatogenesis process and regulates transcription of the c-Kit and Stra8 genes at meiotic entry through specific alterations of histone modifications	Di Giacomo et al. (2016)
Trinitrobenzene sulfonic acid (TNBS)-induced colitis in wildtype and CB2^−/−^ mice	20 mg/kg, i.p. 30 min before the induction of colitis and then twice daily for 3 days	Colitis	Reduces intestinal inflammation via a decrease in colonic adhesions and myeloperoxidase activity	Storr et al. (2009)
Oil of mustard-induced model of colitis in CD-1 mice	20 mg/kg, i.p. 30 min before the induction of colitis and then twice daily for 3 days	Colitis	Reductions in overt inflammatory damage and bowel dysmotility	Kimball et al. (2006)
Dextran sulfate sodium (DSS)- induced colitis in BALB/c mice				
IL-10^−/−^ mice model of colitis	1, 2.5, 5 mg/kg i.p. for 7 weeks	Colitis	Anti-inflammatory activities through inhibiting activated T cells, and inducting apoptosis in T cells	Singh et al. (2012)
Dextran sulfate sodium (DSS)- induced colitis in BL/6 mice				
Oil of mustard-induced model of colitis in CD-1 mice	1 mg/kg s.c	Colitis	Modulation of GI motility attenuating the associated diarrhea	Kimball et al. (2010)
LPS-stimulated transit in Sprague–Dawley rats	1 mg/kg s.c	Colitis	Suppresses GI transit via inhibition of cyclooxygenase	Mathison et al. (2004)
Cerulein-induced acute pancreatitis in WT and MK2^−/−^mice	5 μg/g, i.p. 30 min before the induction of acute pancreatitis	Acute pancreatitis	Suppression of JNK, stimulation of p38 and MK2-signaling pathway reducing the pancreatic injury	Michler et al. (2013)
GalN/LPS-induced acute liver injury in C57BL/6 mice	20 mg/kg i.p, two doses administered 24 and 2 h before the GalN/LPS injection	Acute liver injury	Mediates an M1 to M2 transition in macrophages and modulates the expression of miR-145 to hamper the TLR4 signaling stimulation	Tomar et al. (2015)
Alcohol-fed WT and CB2^_/_^ mice induced fatty liver	3 mg/kg, i.p. for 10 days	Alcoholic liver disease	Anti-inflammatory effects via upregulating of HO-1 in macrophages	Louvet et al. (2011)
Ethanol-fed WT and CB2Mye^−/−^, and ATG5Mye^−/−^ mice	3 mg/kg, i.p. for 10 days	Alcoholic liver disease	Stimulates autophagic process via upregulating of HO-1 in macrophage that mediates the anti-inflammatory and anti-steatogenic activities of CB2 receptors	Denaës et al. (2016)
CCl4-Induced liver cirrhosis in Wistar rats	1 mg/kg, s.c. for 9 days	Liver cirrhosis	Mitigates hepatic fibrosis via decreasing collagen content, α-SMA, and increasing the proteolytic enzyme MMP-2	Muñoz-Luque et al. (2008)
Bile duct ligation (BDL)-induced cirrhotic rats	1 mg/kg, i.p. from days 35–42 days of BDL	Liver cirrhosis	Suppresses mesenteric blood flow leading to mitigation of liver fibrosis	Huang et al. (2012)
Thioacetamide or bile duct ligation-induced cirrhotic rats	1 mg/kg, orally for 2 weeks	Liver cirrhosis	Improves phagocytosis of peritoneal macrophages through suppressing the TNFα signaling, pro-inflammatory cytokines secretion and oxidative stress	Yang et al. (2014)
CCl4-Induced liver cirrhosis in sprague–Dawley rats	10 mg/kg, i.p. 2 h prior to the start of portal pressure measurements	Liver cirrhosis	Mediates HO-1 pathway which decreases vasoconstrictor production and portal hypertension related to PPARγ and CB2R	Steib et al. (2013)
CCl4-Induced liver injury in WT and CB2^_/_^ mice	3 mg/kg, i.p. before CCl4	Liver fibrosis	Mitigates hepatic injury and promotes hepatic regeneration through a paracrine mechanism including hepatic myofibroblasts and antifibrogenic effects	Teixeira-Clerc et al. (2010)
CCL4 plus clodronate- induced liver injury in C57BL/6 mice	10 mg/kg, i.p. before CCl4	Liver fibrosis	Transcriptional regulation of the CB2 receptor gene in hepatocytes by LXRα resulting in inhibition of USP4-stabilizing TβRI through miR-27b	Wu et al. (2019)
Hepatic ischemia/reperfusion in WT and CB2^_/_^ mice	i.p. 60 min prior to the occlusion of the hepatic artery and the portal vein	Hepatic ischemia/reperfusion	Attenuates oxidative stress and the infilteration of inflammatory cells	Bátkai et al. (2007)
Hepatic ischemia/reperfusion in C57BL/6 mice	0.2 mg/kg, i.p. 24 h before the experiment	Hepatic ischemia/reperfusion	Selective depletion or deactivation of HSCs through CB2R activation reduces CD4+ T cell–dependent I/R injury	Reifart et al. (2015)
Collagen-induced arthritis (CIA) mice	1, 4 mg/kg, i.p. from day 15 to day 35	Rheumatoid arthritis	Inhibits production of pro-inflammatory cytokines, and prevents formation of bone-resorbing cells	Fukuda et al. (2014)
Collagen-induced arthritis (CIA) in mice	10 mg/kg, i.p. from day 22 to day 45	Rheumatoid arthritis	Inhibits osteoclastogenesis and inflammation-mediated bone destruction via inhibiting NF-kB signaling pathway	Zhu et al. (2019)
Experimental autoimmune uveoretinitis in B10.RIII mice and BALB/c mice	0.015–1.5 mg/kg, i.p	Autoimmune uveoretinitis	Anti-inflammatory activity through suppressing the stimulation and function of autoreactive T cells and averting leukocyte trafficking into the inflamed retina	Xu et al. (2007)
Hypochlorite-induced systemic sclerosis in BALB/c, C57BL/6 CB2^−/−^ mice	1,1.5, 2, 2.5, 3, and 4 mg/kg, i.p. for 6 weeks	Systemic sclerosis	Inhibits systemic fibrosis, skin fibroblast proliferation and autoimmune reaction	Servettaz et al. (2010)
I/R Injury of albino NMRI mice kidney	0.2, 1 and 5 mg/kg, i.p. 30 min prior initiation of reperfusion-induced ischemia	Renal ischemia reperfusion	Suppression of inflammatory cytokines secretion by NF-κB and mitigates apoptosis	Feizi et al. (2008)
Cyclophosphamide-induced cystitis in C57BL/6J mice	1 mg/kg, i.p. 30 min before cyclophosphamide	Cystitis	Activates autophagy via AMPK-mTOR pathway mitigating bladder inflammatory responses and severity of cystitis	Liu et al. (2020a)
Bleomycin-induced dermal fibrosis in WT and CB2^−/−^ mice	2.5 mg/kg, i.p. for 4 weeks	Dermal fibrosis	⁃Antifibrotic effects by preventing the infiltration of leukocytes into skin lesion	Akhmetshina et al. (2009)
Paraquat-induced lung injury in Sprague-Dawley rats	5 and 20 mg/kg, i.p. before paraquat administration	Lung injury	Mitigates lund injury via suppressing the stimulation of MAPKs and NF-kB signaling	Liu et al. (2014)
Bleomycin-induced pulmonary fibrosis in C57BL/6 mice	2.5 mg/kg, i.p. for 21 days	Pulmonary fibrosis	Anti-fibrotic activity via repressing TGF-β1/Smad2 signaling pathway	Fu et al. (2017)
Nicotine-induced lung fibrosis in swiss mice	1 mg/kg, i.p. before nicotine administration	Pulmonary fibrosis	Anti-fibrotic activity via downregulating the expression of connective tissue growth factor, and α-SMA	Wawryk-Gawda et al. (2018)
Lung ischemia/reperfusion Injury in C57BL/6 mice	5 mg/kg, i.p. 5 min before occlusion	Lung ischemic reperfusion injury	Attenuates the inflammation and oxidative stress relies on activation of PI3K/Akt signaling	Zeng et al. (2019)
Lung ischemia/reperfusion Injury in C57BL/6 mice	5 mg/kg, i.p. 5 min before occlusion	Lung ischemic reperfusion injury	Inhibits oxidative stress via downregulation of NOX2	Huang et al. (2020)
Respiratory syncytial virus challenged Balb/c mice	i.p. for 5 days	Acute respiratory tract infections	Anti-inflammatory activity via reducing the influx of BAL cells, leukocyte migration into the lungs, and cytokines/chemokines	Tahamtan et al. (2018)
Skeletal muscle contusion model in Sprague-Dawley rat	10 mg/kg, i.p. injected 30 min after contusion and once a day for 13 days	Skeletal muscle contusion	Inhibits fibrosis and improves muscle regeneration via reducing TGF-β1, fibronectin-EIIIA and α-SMA, decreases production of myofibroblasts, and concurrently upregulation of MMP-1/2	Yu et al. (2015)
Incised skin wound model in BALB/c mice	3 mg/kg, i.p. for 1–9 days	Skin wound healing	Inhibition of inflammatory process by attenuating infiltrated M1 macrophage cells and enhancing M2 macrophage phenotype	Du et al. (2018)

**TABLE 3:** Pharmacological activities, mechanism and therapeutic potential of JWH133 in the *in vitro* studies.

Experimental model	JWH133 concentration	Indication/Disease	Demonstrated actions and mechanisms	References
Plasmacytoid dendritic cells stimulated with CpGODN Type A 2216	0.001, 0.01, and 0.1 μM	Inflammation	Suppresses CpG-stimulated IFNα and TNFα dependent on modifying the phosphorylation of AKT	Henriquez et al. (2019)
LPS/IFN-γ or Theiler’s virus (TMEV)-activated macrophages	10 nM	Inflammation	Inhibits IL-12p40 production and enhances IL-10 biosynthesis via activation of ERK1/2 MAP kinase	Correa et al. (2005)
	100 nM, 1 μM, and 5 μM			
Human coronary artery endothelial cells (HCAECs) activated with TNF-α	0.5, 2.5, and 4 μM	Atherosclerosis	Attenuates TNF-α-triggered NF-κB and RhoA activation, upregulates of adhesion molecules ICAM-1 and VCAM-1, decreases expression of monocyte chemoattractant protein, TEM of monocytic THP-1 cells, and monocyte-endothelial adhesion	Rajesh et al. (2007)
Human coronary artery smooth muscle cells (HCASMCs) activated with TNF-α	0.5–4 μM	Atherosclerosis	Mitigates the activation of induced Ras, mitogen-activated protein kinases (p38 MAPK, ERK ½), stress-activated protein kinases (SAPK)/Jun amino-terminal kinases (JNK) and Akt	Rajesh et al. (2008)
Human neutrophils	0.3 and 1 μM	Atherosclerosis	Suppresses neutrophil production of MMP-9 via attenuation of ERK1/2 phosphorylation	Montecucco et al. (2012)
Normal-cultured and oxidative low-density lipoprotein (OxLDL)-loaded RAW264.7 and primary macrophages	0.1, 1, and 10 μM	Atherosclerosis	Improves efferocytosis via increasing expression of tyrosine kinase family phagocytic receptors, inhibition of RhoA GTPase stimulation, and alleviation of oxidative/inflammation responses	Jiang et al. (2016)
Human osteoblastic hFOB 1.19 cells	1, 2, 5	Osteoporosis	Osteogenic differentiation mediated by CB2R dependent mechanism involved autophagy activation and p62- mediated Nrf2 degradation	Xu et al. (2020)
	10, and 20 μM			
Methylprednisolone-induced osteoclast overactivity from healthy donors	100 nM from day 14 to day 21	Osteoporosis	Reduces bone resorption dependent on PKC βII signaling	Bellini et al. (2017)
MDA-MB231 and MDA-MB468 cells	0.1–10 μmol/L	Breast cancer	Inhibits cell proliferation and migration	Qamri et al. (2009)
Rat glioma C6 cell	100 nM	Brain cancer	Induces apoptosis via ceramide synthesis and ERK1/2 activation	Sánchez et al. (2001)
Human umbilical vein endothelial cells (HUVECs)	25 nM	Brain cancer	Direct inhibition of vascular endothelial cell migration and survival as well as the decrease of the expression of proangiogenic factors (VEGF and angiopoietin-2) and MMP-2 in the tumors	Blázquez et al. (2003)
Glioma stem-like cells and glioma cell lines U87MG and U373MG	30 nM	Brain cancer	Stimulates glia cell differentiation in a CB2R-related mechanism	Aguado et al. (2007)
A2058 melanoma cells	10 μM for 4 h	Brain cancer	Reduces adhesion and transmigration of melanoma cells through the cerebral endothelium	Haskó et al. (2014)
A549 cells and HUVECs	10^−4^–10^–8^ mol/l	Lung cancer	Anti-proliferative and anti-angiogenic potential	Vidinský et al. (2012)
			Downregulates MMP-2 activity	
A549 cells co-cultured with huvec	3 μM	Lung cancer	Increases tissue inhibitor of matrix metalloproteinases-1 (TIMP-1) production from lung cancer cells and a consequent stimulation of ICAM-1 expression, thereby modifying the tumor cells microenvironment and inhibiting the angiogenesis	Ramer et al. (2014)
Human lung macrophage stimulated with LPS	1 μM	Lung cancer	Modulates tumor vascularization via reduction of macrophage-derived angiogenic and lymphangiogenic factors	Staiano et al. (2016)
T-ALL patients and Jurkat cell line	100 nM	Leukemia	Anti-proliferative, pro-apoptotic and cell cycle arrest	Punzo et al. (2018a)
ARO/IL-12, ARO and ARO/CB2 thyroid carcinoma cells	2 μM for 24 h	Thyroid carcinoma	IL-12-mediated CB2 upregulation rendered the thyroid cancer cells more responsive to CB2 agonist-induced apoptosis and remission of the tumors	Shi et al. (2008)
Saos-2, MG-63, MNNG/HOS, KHOS/NP, Hs888Lu and U-2 OS Osteosarcoma cells	100 nM for 24 h	Osteosarcoma	Anti-proliferative, pro-apoptotic, anti-invasive effect with downregulation of Notch-1 and MMP-2	Punzo et al. (2017)
Isolated perfused rat hearts subjected to 30 min global ischemia followed by 120 min reperfusion	1,10, and 100 nmol/L for 15 min before I-R treatment	Myocardial infarction	Increases phosphorylated ERK1/2 and preventing MPTP opening	Li et al. (2014)
Adult cardiac myocytes from WT or CB2/mice	1 µM	Myocardial infarction	Prevention of oxidative stress-induced cardiac myocyte and fibroblast apoptosis and the suppression of myofibroblast activation	Defer et al. (2009)
Mice cardiomyocytes under oxygen-glucose deprivation (ODG)	1, 10, and 100 nM 10 min before OGD challenge	Myocardial infarction	Modulation of NLRP3 inflammasome pathway	Yu et al. (2019)
Mouse RAW264.7 macrophages and 3T3-L1 fibroblasts	1 or 3 μM for 24 h	Obesity	Attenuates pro-inflammatory M1 macrophage cytokines through the Nrf2/HO-1 mechanism	Wu et al. (2020)
Obese-derived white adipocyte (ADP)	100 nM	Obesity	Mitigates the obesity-associated inflammation, and the excess lipid storage in white adipose tissue WAT through modulating perilipin expression, up-regulating IL-4, and stimulating UCP-1 signaling	Rossi et al. (2016)
Rat m5F insulinoma β-cells	10^–6^ M	Diabetes mellitus	CB2R stimulation is linked to Ca2+ mobilization from the endoplasmic reticulum stores leading to insulin release in pancreatic β-cells	De Petrocellis et al. (2007)
Isolated uterus from female ICR mice stimulated with exogenous PGE2	10^−8^–10^–5^ M, for 20 min	Female reproduction	Mitigation of myometrial contractility dependent on the suppression of prostaglandin release/synthesis	Pagano et al. (2017)
SPG germ cells obtained from testes of immature 7-day-old swiss CD-1 mice	10^−6^ M for 0–60 min	Spermatogenesis	Pro-differentiated effect via induction of the phosphorylated ERK 1/2 MAPK in spermatogonia and their progression toward meiosis	Grimaldi et al. (2009)
SPG germ cells obtained from testes of immature 7-day-old swiss CD-1 mice	1 µM for 24 h	Spermatogenesis	Accelerates the spermatogenesis process and regulates transcription of the c-Kit and Stra8 genes at meiotic entry through specific alterations of histone modifications	Di Giacomo et al. (2016)
Mucosal samples from areas of inflamed/uninflamed colon from IBD patients and Caco-2 cell line	10 µM for 6 h	Colitis	Enhances colon cells proliferation and migration and affects secretome characteristics that facilitate mucosal healing	Tartakover Matalon et al. (2020)
Isolated ileum from Sprague-Dawley rats injected with LPS	10^–2^ M	Colitis	Reduces the accelerated contraction induced by LPS via downregulation of the FOS expression in enteric glial and neurons	Duncan et al. (2008)
RAW264.7 macrophages activated with LPS	5 μM for 24 h	Alcoholic liver disease	Anti-inflammatory effects via upregulating of HO-1 in macrophages	Louvet et al. (2011)
RAW264.7 macrophages from CB2Mye^−/−^ mice activated with LPS	5 μM for 6 h	Alcoholic liver disease	Stimulates autophagic process in macrophage mediated the anti-inflammatory and anti-steatogenic activities of CB2R	Denaës et al. (2016)
Isolated kupffer cells activated with zymosan A and LPS	5 μM for 3 h	Liver cirrhosis	Mediates HO-1 pathway which decreases vasoconstrictor production and portal hypertension related to PPARγ and CB2R	Steib et al. (2013)
Cultured Th17 lymphocytes	5 μM	Liver fibrosis	Decreases IL-17 production by Th17 lymphocytes relies on STAT5 pathway, and by dampening the proinflammatory activity of IL-17, while conserving IL-22 production	Guillot et al. (2014)
IL-17-induced inflammatory				
Response on macrophages and hepatic myofibroblasts				
AML12 cells exposed to TGF-β1	1, 3, and 10 μM for 1 h	Liver fibrosis	Transcriptional regulation of the CB2 receptor gene in hepatocytes by LXRα that in turn inhibits USP4-stabilizing TβRI through miR-27b	Wu et al. (2019)
Human liver sinusoidal endothelial cells (HLSECs) treated with TNF-α	0–4 μM for 4 h	Hepatic ischemia/reperfusion	Mitigates the TNF-α-stimulated ICAM-1 and VCAM-1 expression and decreases the adhesion of human neutrophils	Bátkai et al. (2007)
Fibroblast-like synoviocytes activated with TNF-α	1, 10, and 50 μM for 24 h	Rheumatoid arthritis	Inhibits production of pro-inflammatory cytokines, and prevents formation of bone-resorbing cells	Fukuda et al. (2014)
Bone marrow-derived macrophages cultured with TNF-α	1 μM for 24 h	Rheumatoid arthritis	Inhibits osteoclastogenesis and inflammation-mediated bone destruction via inhibiting NF-kB signaling pathway	Zhu et al. (2019)
Mesenchymal stromal cells from ITP patients	2.5 μM for 24 h	Immune thrombocytopenia	CB2 stimulation attenuates apoptosis via Bcl-2 signaling, and restores the immune-modulatory properties of MSCs	Rossi et al. (2019a)
Mice lung fibroblasts exposed to TGF-β1	10 μM for 48 h	Pulmonary fibrosis	Inhibited firbosis via repressing TGF-β1/Smad2 signaling pathway	Fu et al. (2017)
Human Adipose tissue mesenchymal stromal cells (atMSCs)	1, 3, 10, and 30 μM	Wound healing	Enhances secretion of VEGF, TGF-β1 and HGF, which in turn enhances the regenerative activity of at MSCs	Ruhl et al. (2020)
Mesenchymal stem cells	3 μM for 1 h or 6 h	Bone healing	Induction of p42/44 MAPK that mediates migration of mesenchymal stem cells	Schmuhl et al. (2014)
Human Tenon’s fibroblasts exposed to TGF-β1	0.5 μM for 24 h before TGF-β1	Wound healing	Suppresses ECM synthesis and MAPKs (ERK1/2, p38, and JNK) induced by TGF-β1 and reduces the contractility of HTFs	Guan et al. (2017)
Corneal epithelial cells	300 nM	Wound healing	Exerts chemorepulsive activity	Murataeva et al. (2019)
			Stimulates *p*-ERK and cAMP production	
Differentiating oligodendrocyte progenitor cells	0.1, 0.5, and 1 µM for 48 h	Brain repair	Enhances oligodendrocyte differentiation dependent on stimulation of *p*-Akt and mTOR signaling	Gomez et al. (2011)

Collectively, the modulation of CB2R signaling represents a promising, nonpsychoactive pharmacological target that can be harnessed to treat a wide number of disorders. This review emphasizes the polypharmacological properties and therapeutic potential of JWH133, its molecular mechanism, and signaling pathways in different pathological conditions except neuronal diseases as the neuroprotective effects of JWH133 are discussed in another review. The neuroprotective role of JWH133 has been well demonstrated in a large number of experimental studies and is not included in the present study.

## Therapeutic Potential of JWH133

### JWH133 in Inflammation

**FIGURE 2:**
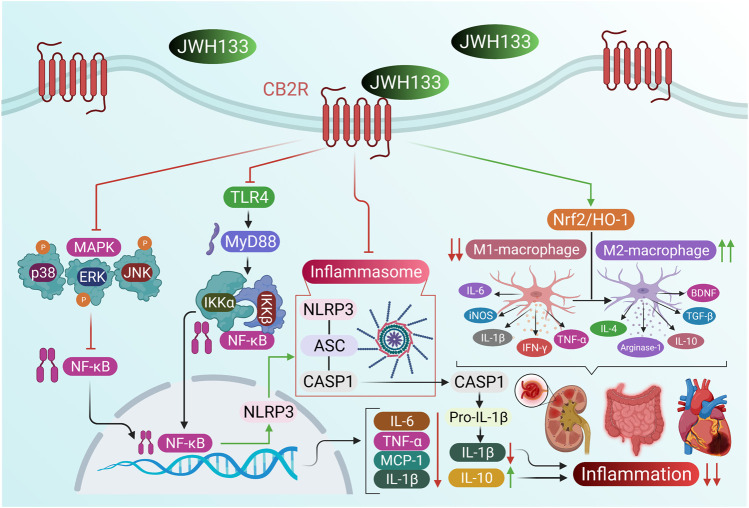
The anti-inflammatory activity and mechanisms of JWH133.

Increasing evidence suggests that CB2R stimulation has anti-inflammatory effects in various inflammatory diseases (Storr et al., 2009; Hao et al., 2010; Gui et al., 2015). CB2R stimulation also inhibits the production of inflammatory cytokines and chemokines and induces the secretion of anti-inflammatory cytokines (He et al., 2019). Indeed, CB2R-deficient mice have an exaggerated inflammatory response (Turcotte et al., 2016). Thus, therapeutic approaches that target the modulation of CB2R signaling might hold promise for the treatment of inflammatory pathologies. The anti-inflammatory activity and mechanisms of JWH133 are displayed in Figure 2.

Local application of JWH133 to the joints of normal rats induced a dose-dependent increase in synovial blood flow. This effect was abolished by pretreatment with AM630 or the TRPV1 antagonist SB366791, indicating that TRPV1 is necessary for CB2R-mediated activity (McDougall et al., 2008). CB2R primarily localizes on immunocytes, suggesting that these cells mediate the vasomotor activities of JWH133. However, JWH133-induced vasodilation was markedly reduced in acute and chronically arthritic knees, suggesting that the expression and sensitivity of articular CB2R are altered in inflamed joints. CB2R activation in the knee joint may yield vasodilation via vanilloid TRPV1 channels. Further studies are needed to characterize the molecular and biochemical pathways linking TRPV1 and CB2R.

Plasmacytoid dendritic cells (pDC) play a pivotal role in initiating host immunity. Enhanced and chronic pDC stimulation is a characteristic of autoimmune disorders such as systemic lupus erythematosus and rheumatoid arthritis (Colonna et al., 2004). Treatment of pDC with JWH133 suppressed CpG-stimulated IFNα and TNFα responses (Henriquez et al., 2019). JWH133 also suppressed key markers of pDC stimulation, including phosphorylated levels of IRF7, TBK1, NFκB, and IKKγ. Similarly, AKT phosphorylation at S473 and T308 was differentially modified by treatment with JWH133. Thus, CB2R activation represents a potential target for treating inflammatory pathologies caused by aberrant pDC activity.

Tissue mast cells are involved in several inflammatory conditions and play a key role in multiple sclerosis and rheumatoid arthritis (Puxeddu et al., 2003). In a mouse model, JWH133 injection reversed inflammation induced by injecting the compound 48/80 into the ear pinna (Jonsson et al., 2006). Interpreting the CB2-agonist effect of JWH133 is complicated because CB2R antagonism by SR144528 also produced an anti-inflammatory effect in mice. *In vitro* results were discordant because JWH133 did not affect mast cell degranulation induced by compound 48/80 in mouse skin slices, perhaps owing to an unrecognized CB2R deficiency in the skin of the experimental mouse strain. JWH133 also failed to inhibit [3H] pyrilamine binding to histamine H1 receptors *in vitro*. Therefore, the capacity of JWH133 to influence mast cell-mediated inflammatory responses *in vivo* could be mediated by an indirect action on the mast cells.

In a rat model of cecal ligation and puncture (CLP)-induced polymicrobial sepsis, JWH133 reduced injury in the brain, heart, lung, and liver and attenuated the expression of caspase-3, p-NF-κB, TNF- α, IL-1β, and IL-6 levels while enhancing the expression of the anti-inflammatory cytokine IL-10 levels (Çakır et al., 2020). Thus, CB2R activation reduced inflammatory mediator expression by inhibiting apoptotic and NF-κB signaling, suggesting that JWH133 has therapeutic benefit in sepsis. JWH133 suppressed LPS/IFN-γ or Theiler’s virus -activated macrophage-mediated IL-12p40 release in a dose-dependent manner, whereas cotreatment with SR-144528 reversed this effect (Correa et al., 2005). The effect of JWH133 on IL-12p40 release was mediated by ERK1/2 signaling, as indicated by a significant increase in ERK1/2 kinase. Suppression of ERK1/2 by the selective inhibitor PD98059 amplified LPS-induced IL-12p40 release, suggesting that persistent stimulation of ERK1/2 inhibits the release of IL-12p40. CB2R stimulation by JWH133 boosted IL-10 release from LPS/IFN-γ-stimulated macrophages. The effect was abrogated by SR144558 or ERK inhibitor PD98059. Blocking IL-10 with neutralizing antibody led to enhanced IL-12p40 production by LPS-activated macrophages in the absence or presence of JWH133, suggesting that endogenous IL-10 is involved in mediating the inhibitory effect of JWH133 on IL-12p40 secretion by activated macrophages. Thus, CB2R specific ligands could be beneficial for treating chronic inflammatory disorders.

In a carrageenan-induced inflammatory model, systemic administration of JWH133 3 h after carrageenan markedly ameliorated ipsilateral hindpaw weight-bearing and paw volume (Elmes et al., 2005). Similarly, pretreatment with JWH133 had the same effect on weight-bearing. The post-treatment effects of JWH133 on weight-bearing and paw volume were analogous to the systemic post-treatment effects of morphine and rofecoxib. Thus, CB2R activation by JWH133 mitigated inflammatory reaction and swelling, indicating that CB2R agonists might be a beneficial target for treating inflammatory pain responses. In contrast, JWH133 increased intracellular Ca^2+^ levels in human retinal pigment epithelial cells, indicating their responsiveness to JWH133 (Hytti et al., 2017). However, JWH133 did not inhibit oxidative stress-induced apoptosis mediated by reactive aldehyde 4-hydroxynonenal. Furthermore, JWH133 triggered cell death and increased the production of proinflammatory cytokines IL-6 and IL-8 via an ERK1/2-related mechanism. Contrary to the previous findings, CB2R activation increased inflammation instead of reducing it in human retinal pigment epithelial cells.

### JWH133 in Atherosclerosis

Atherosclerosis is a chronic inflammatory disease and the leading cause of cardiac disorders and stroke worldwide (Libby, 2002). A significant link has been established among inflammatory processes, oxidative stress, nitrosative stress, and fat metabolism in the pathophysiology of atherosclerosis and vascular remodeling after injury (Patel et al., 2000; Hansson and Libby, 2006). The cannabinoid system has been identified to be associated with a growing number of chronic inflammatory diseases such as atherosclerosis (Pacher et al., 2006; Pacher and Mechoulam, 2011). CB2R stimulation has been specifically proposed to regulate atherosclerosis (Steffens et al., 2005). In this latter study, oral treatment with low-dose D9-tetrahydrocannabinol (THC, 1 mg kg^−1^ per day) markedly decreased plaque development in ApoE-knockout mice. Another study showed that administration of a CB2R/CB1R agonist ameliorated atherosclerosis in ApoE-deficient mice via a proposed CB2R-dependent mechanism (Zhao et al., 2010). TNF-α activates NF-κB and RhoA and upregulates adhesion molecules ICAM-1 and VCAM-1 in human coronary artery endothelial cells (HCAECs), thereby enhancing the expression of monocyte chemoattractant protein and promoting transendothelial migration of monocytes and monocyte–endothelial cell adhesion (Rajesh et al., 2007). All these effects were mitigated by pretreating HCAECs with JWH133.

JWH133 attenuated TNF-α- and/or endotoxin-induced expression of ICAM-1 and VCAM-1 in isolated aortas and prompted monocyte-aortic vascular endothelium adhesion. The protective effect of JWH133 was abolished by CB2R blockers (SR-144528 and AM-630) but not by CB1R (SR-141716 and AM-251) blockers. Thus, CB2R stimulation might alleviate endotoxin-driven vascular inflammation. Similarly, pretreatment of human coronary artery smooth muscle cells with JWH133 resulted in dose-dependent inhibition of proliferation and migration of vascular smooth muscle cells, which was reversed by SR2/AM630 but not by the CB1 blocker SR1 (Rajesh et al., 2008). Moreover, JWH133 mitigated the TNF-α activation of Ras, MAPKs (p38 and ERK 1/2), stress-activated protein kinases (SAPK)/Jun amino-terminal kinases (JNK), and Akt. These effects were abolished by AM630, indicates that CB2R activation counteracted TNF-α-induced pathways.

In another study, JWH133 significantly decreased MMP-9 content in ApoE2/2 mouse aortic root and carotid plaques (Montecucco et al., 2012). *In vitro*, preincubation of human primary neutrophils with JWH133 significantly reduced TNF-α-induced MMP-9 release, and this effect was abrogated by coincubation with AM630. The CB2R-mediated protective effect occurred via attenuation of TNF-α-induced ERK1/2 phosphorylation. Because CB2R stimulation suppressed neutrophil production of MMP-9 *in vivo* and *in vitro*, this treatment strategy could specifically diminish carotid atherosclerotic susceptibility in humans.

JWH133 induced dose-dependent phagocytosis of apoptotic cells in normal-cultured and oxidative low-density lipoprotein (OxLDL)-loaded RAW264.7 and primary macrophages (Jiang et al., 2016). JWH133 also induced the expression of tyrosine kinase family phagocytic receptors MerTK, Tyro3, and Axl. Efferocytosis of macrophages is mainly mediated by tyrosine kinase family phagocytic receptors (Seitz et al., 2007). JWH133 also decreased OxLDL-induced TNF-α and reactive oxygen species (ROS) production and blocked RhoA GTPase stimulation. Thus, selective CB2R activation improved efferocytosis of normal-cultured and OxLDL-loaded macrophages via induction of the tyrosine kinase family phagocytic receptors, inhibition of RhoA GTPase stimulation, and alleviation of oxidative/inflammation responses, thereby reducing the risk and promoting the stability of atherosclerotic plaques. Administration of JWH133 to ApoE/− mice fed on a high-cholesterol diet caused significant reduction of proliferation, decreased smooth muscle cell content, and reduced macrophage infiltration (Molica et al., 2012). Complete endothelial repair was observed after 14 days in both JWH133 and vehicle-treated mice, indicating that the CB2 agonist does not inhibit endothelial repair. CB2 deficiency resulted in increased intima formation compared with WT, whereas JWH133 did not affect intimal formation in CB2^−/−^ mice. Genetic CB2R deletion increases neointima formation and *in situ* apoptosis after carotid balloon injury; enhances macrophage adhesion and migration; and enhances smooth muscle cell proliferation *in vitro*. In conclusion, pharmacological activation or genetic deletion of CB2R modulates neointima formation via smooth muscle cells and macrophages. Treatment of ApoE^−/−^ hypercholesterolemic mice with JWH133 mitigated ROS release and NADPH-oxidase expression in mice penis (Fraga-Silva et al., 2013).

Furthermore, JWH133 upregulated endothelial NO synthase in the corpus cavernosum and increased nitric oxide bioavailability. The reduction in oxidative stress levels was associated with a decrease in collagen content. Therefore, CB2R stimulation attenuated ROS production and fibrosis associated with erectile dysfunction in hypercholesterolemic mice. In contrast, intraperitoneal injections of JWH133 in LDLR^−/−^ mice on a high-cholesterol diet resulted in no significant difference in intimal lesion size in sections of the aortic roots and arches, indicating that CB2R stimulation did not modulate atherogenesis in mice (Willecke et al., 2011). Further, JWH133 treatment did not mitigate the contents of lipids, macrophages, collagen, T cells, and smooth muscle cells and the rate of cell apoptosis in atherosclerotic mice. However, JWH133 reduced intraperitoneal macrophage numbers after 72 h of intraperitoneal injection in a model of thioglycollate-induced peritonitis but not after 4 h. Neither genetic deficiency nor pharmacologic stimulation of the CB2R caused a change in the expression of proinflammatory cytokines (IL-6, MCP-1, IL-10, IFNγ, or IL-12p70) in mice challenged with intraperitoneal TNF-α or inflammatory cell adhesion in murine endothelial cells isolated from LDLR^−/−^ mice. Therefore, neither CB2R activation nor its genetic deficiency modulated atherogenesis.

### JWH133 in Bone Disorders

CB2Rs are highly expressed in bone cells compared with CB1Rs and have a crucial role in controlling the balance between bone resorption and osteogenesis (Whyte et al., 2012). CB2Rs are upregulated during bone remodeling (Idris, 2012). CB2R activation improves osteoblast proliferation and function by enhancing the expression of osteogenic factors such as RUNX2, bone sialoprotein, osteopontin, alkaline phosphatase, and osteocalcin (Qian et al., 2010).

Preclinical studies revealed that CB2R-knockout mice developed osteoporosis at 12 months, reduced osteoblast production and function, and enhanced osteoclast production (Sophocleous et al., 2011). Clinical studies of postmenopausal women demonstrated that the gene encoding the CB2R (*CNR2*) is responsible for low bone mineral density (Zheng et al., 2019). Thus, CB2Rs may be a translational target for pharmacologic agents that augment bone regeneration, but quality clinical trials are warranted.

#### Osteoarthritis

In developed nations, osteoarthritis (OA) is the most common chronic joint disease with a social cost of approximately 0.5% of gross domestic product (Puig-Junoy and Ruiz Zamora, 2015). It is marked by pain and frequent disability and is correlated with anxiety, depression (Axford et al., 2010), and cognitive changes (Moriarty et al., 2011). Spinal CB2R expression is correlated with knee joint damage (macroscopic chondropathy score) in human post mortem samples (Burston et al., 2013). Systemic administration of JWH133 mitigated OA pain induced by monosodium iodoacetate, decreased the expression of inflammatory cytokines (IL-1β and TNFα), and increased the levels of anti-inflammatory IL-10. Spinal administration of JWH133 suppressed noxious mechanically evoked responses of spinal neurons in animal model of OA pain, but not in naive rats, indicating great potential of this treatment route. SR144528 abrogated the effect of JWH133. Systemic administration reduced the expression of glial fibrillary acidic protein (GFAP; a marker of reactive gliosis) and MMP-2 and MMP-9 in the spinal cord. These findings suggest that CB2R stimulation diminished central sensitization, thereby mitigating pain behavior.

In another study, JWH133 improved the alternations in nociception and anxiety behaviors but did not ameliorate memory impairment in an animal model of OA pain (La Porta et al., 2015); this was probably owing to a direct result of the pain-relieving effect mediated by CB2R. The absence of a memory-protective effect suggests that the JWH133-mediated improvement of these symptoms is owing to the direct effect of JWH133 on emotion and cognition.

#### Osteoporosis

Osteoporosis is a systemic skeletal disease characterized by low bone mass, damage of bone tissue, and decreased bone mineral density and is considered a silent disease until a fracture occurs (Pisani et al., 2016). In human osteoblast hFPB1.9 cells, JWH133 produced a dose-dependent increase in autophagy, as measured by the conversion of LC3I to LC3II, increased beclin-1 expression, and enhanced p62 degradation (Xu et al., 2020). Furthermore, JWH133 inhibited mTOR signaling by reducing the levels of phosphorylated mTOR, P70S6K, and 4EBP1 in hFOB 1.19 cells. However, CB2R-knockdown abrogated the effect of JWH133 on autophagy. JWH133 also increased alkaline phosphatase activity and bone mineralization and increased the expression of osteogenic markers osteopontin and osteocalcin. Interestingly, the osteogenic activities mediated by CB2R stimulation were significantly attenuated by the autophagic inhibitor 3-MA, indicating that the stimulation of autophagy is needed for CB2R-mediated osteoblast differentiation. Moreover, JWH133 decreased nuclear Nrf2 accumulation and upregulated Keap1 and re-expression of p62 prevented CB2R agonist-mediated Nrf2 deactivation. In summary, osteogenic differentiation mediated by CB2R involves autophagy activation and p62-mediated Nrf2 degradation.

Antagonism of vanilloid receptor 1 (TRPV1) and/or activation of CB2R reduces the number and activity of osteoclast cells (Rossi et al., 2019b). Methylprednisolone-induced telomerase activity was markedly decreased by JWH133 and the TRPV1 antagonist I-RTX in healthy subject-derived osteoclasts (Bellini et al., 2017). Additionally, JWH133 and I-RTX reverted methylprednisolone-induced osteoclast hyperactivity, evidenced by a significant reduction in osteoclast numbers. Furthermore, CB2R activation by JWH133 hampered resorption and modulated protein kinase C beta II (PKC βII) signaling induced by methylprednisolone, suggesting that JWH133 reduced PKC βII signaling-dependent bone resorption. Conversely, JWH133 stimulated osteoclast formation in mouse osteoblast–bone marrow cocultures (Idris et al., 2008). It produced a dose-dependent increase in RANKL-induced osteoclast formation and increased osteoclast size and nuclearity with no remarkable effect on apoptotic cell death. The conflicting results on bone resorption and osteoclast function require further investigation.

### JWH133 in Cancer

**FIGURE 3:**
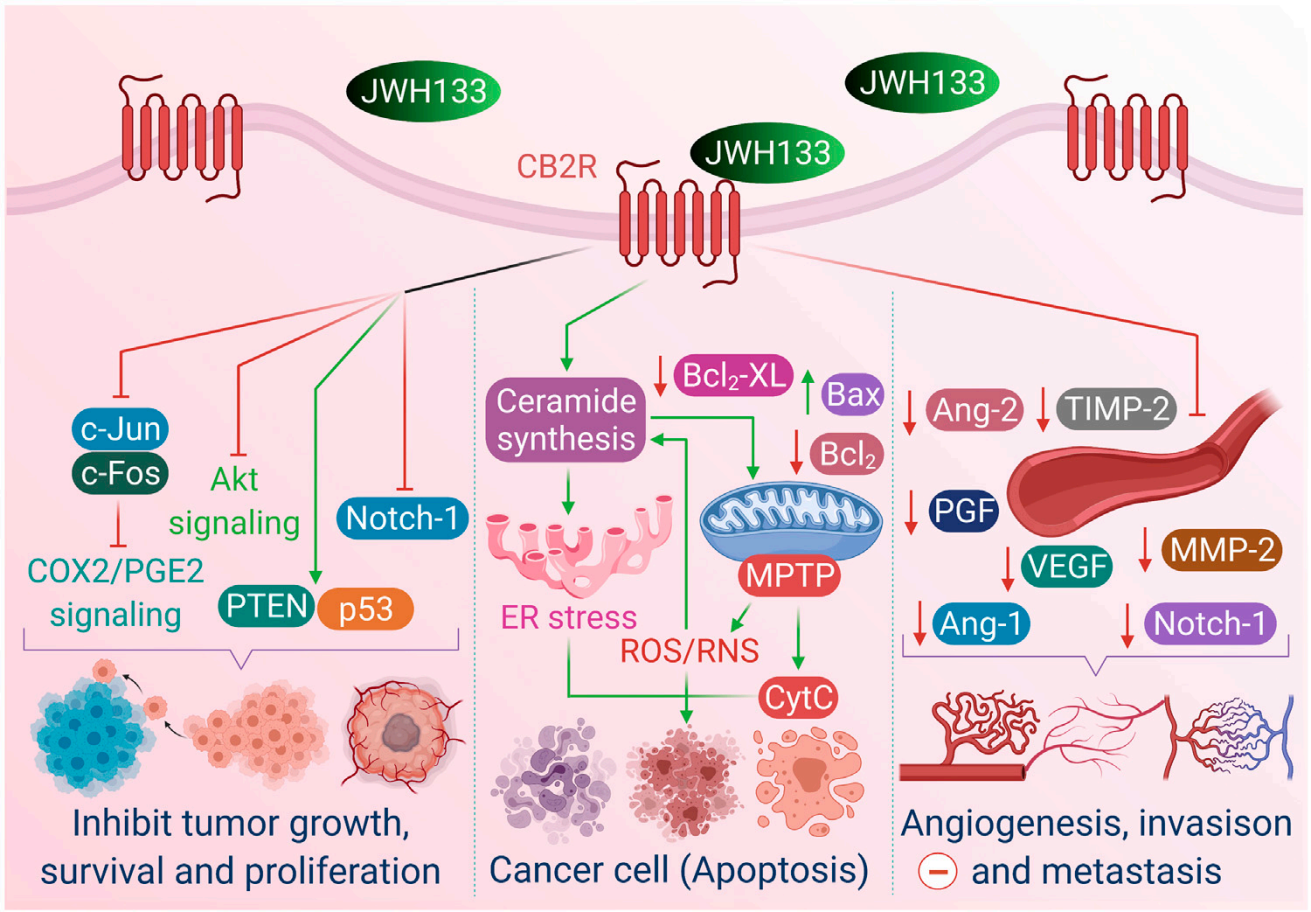
The anticancer properties, effects and mechanism of JWH133.

A previous study suggested that endocannabinoids possess anticancer activity by demonstrating that oral administration of D9-THC, D8-THC, and cannabinol prevented the proliferation of Lewis lung adenocarcinoma cell growth *in vitro* and *in vivo* (Munson et al., 1975). Many other cannabinoids have since been demonstrated to inhibit proliferation, metastasis, angiogenesis, and apoptosis in different cancer types *in vitro* and *in vivo* (Casanova et al., 2003; Carracedo et al., 2006; Cianchi et al., 2008). Growing evidence suggests that the anticancer effects of phyto-, endo-, and synthetic cannabinoids are attributed to their ability to modulate cellular signaling mechanisms controlling cell proliferation and survival (Guzmán, 2003; Bifulco et al., 2008). The anticancer properties, effects and mechanisms of JWH133 are presented in Figure 3.

#### Breast Cancer

Breast cancer is the most prevalent cancer type, constituting approximately 30% of newly diagnosed cancers yearly. Almost one-third of breast cancers overexpress the ErbB2 tyrosine kinase receptor (Her2 in humans, Neu in rats) (Baselga and Swain, 2009). Qamri et al. (2009) showed that JWH133 provided a dose-dependent inhibition of the proliferation and migration of MDA-MB231 and MDA-MB468 cells. JWH133 resulted in a significant decrease in tumor growth and lung metastasis and markedly attenuated angiogenesis in mice. However, these effects were abolished by SR144528, suggesting that the anticancer activities were mediated by CB2R. CB2R activation by JWH133 also delayed and reduced mammary gland tumor growth in a PyMT transgenic mouse model by modulating COX-2/prostaglandin E2 signaling. COX-2 expression was inhibited by the downregulation of transcription factors *c-Fos* and *c-Jun* by JWH133 in breast cancer cells. Synthetic cannabinoids may block tumor growth by inducing cell cycle arrest and apoptosis in human breast cancer tumors. Therefore, CB2Rs might provide a clinical therapeutic approach for treating breast cancer proliferation and metastasis.

Caffarel et al. (2010) reported that JWH133 mitigated tumor growth, tumor number, and the severity of lung metastases in MMTV-neu mice, a clinically relevant model of ErbB2-driven metastatic breast cancer. JWH133 inhibited tumor cell proliferation, as indicated by a decreased number of Ki67-positive cells in cannabinoid-treated tumors, stimulated apoptosis in cancer cells by inducing caspase 3, and prevented angiogenesis. In addition, JWH133 induced a CB2R-dependent reduction in N202.1A cell proliferation and xenograft growth. The effect of JWH133 was blocked by SR144528 but not by SR141716, demonstrating the CB2R specificity of JWH133 and subsequent suppression of the protumorigenic AKT pathway.

Low micromolar concentrations of JWH133 decreased the cell viability of MDA-231, 4T1 and MCF7 (Sophocleous et al., 2015). However, nanomolar concentrations augmented human and mouse breast cancer cell-mediated osteoclastogenesis and enhanced osteolysis, and these effects were reversed by CB2-knockout or treatment with AM630, indicating that inactivation of CB2R suppressed osteoclastogenesis in bone metastasis. In addition, JWH133 did not impact osteoblast differentiation in the presence of breast cancer cells-conditioned medium. In contrast, it increased osteoblast differentiation induced by parathyroid hormone, and the ability to stimulate osteoclast formation supported the fact that CB2R stimulation enhanced osteoblast differentiation in a metastatic environment. Furthermore, JWH133 promoted PI3K/AKT activity in a CB2-specific mechanism in the presence of osteolytic and osteoblastic factors such as RANKL and parathyroid hormone. These findings suggest that breast cancer and bone cells respond differently to CB2R agonists depending on cell type and concentration.

#### Colon Cancer

Colon cancer is the second leading cause of cancer mortality in developed nations and the fourth worldwide, with greater than one million newly diagnosed patients yearly (Jemal et al., 2009a).

JWH133 inhibited the adrenaline-driven migration of SW480 colon and MDA-MB-468 breast cancer cells and attenuated T lymphocyte migration induced by chemokine stromal cell-derived factor 1. This effect was not diminished by the selective CB1R agonist docosatetraenoyl ethanolamide (Joseph et al., 2004).

Martínez-Martínez et al. (2016) reported that sub-micromolar doses of JWH133 enhanced cell proliferation of the human colon cancer cell lines HT29, SW480, and LS174T and in nude mice by stimulating the AKT/Protein kinase B pathway. Consequently, JWH133 activated AKT, which induced the phosphorylation and suppression of glycogen synthase kinase-3β (GSK3β), leading to a more aggressive cell phenotype with increased levels of SNAIL, the Snail family zinc-finger transcription factor which induces the initiation of the epithelial–mesenchymal transition (Bachelder et al., 2005) as well as downregulation of E-cadherin and β-catenin delocalization from the cell membrane. Cumulatively, CB2 stimulation with submicromolar concentrations of JWH133 activated PI3K/AKT signaling, thereby promoting colon tumor cell proliferation and aggressiveness. These results must be considered when exploring cannabinoid therapy for patients with colon cancer because of the dose-dependent response and the challenges of delivering the drug to the tumor site.

#### Brain Cancer

Malignant gliomas are considered the most common malignant brain tumors with poor prognosis (Maher et al., 2001). The first study to investigate the antitumor activity of JWH133 was conducted by Sánchez et al. (2001), who demonstrated that incubating rat glioma C6 cells with JWH133 significantly reduced cell viability by approximately 50% owing to the activation of apoptotic cell death via ceramide synthesis and ERK1/2 stimulation. Intratumoral administration of JWH133 in a Rag-2^−/−^ mouse model of glioma resulted in a remarkable reduction in tumor growth by approximately 71%. This antitumor effect was abrogated by SR144528 but not by SR141716. JWH133 prevented the growth of highly malignant human astrocytoma in Rag-2^−/−^ mice. Cumulatively, CB2R activation exerted antitumor activity by inducing apoptosis via ceramide synthesis and ERK1/2 activation.

In a similar study by the same group using the same mouse model, intratumoral treatment with JWH133 significantly downregulated the expression of proangiogenic factors, including vascular endothelial growth factor (VEGF) and angiopoietin 2 (Ang 2), revealing another significant feature of JWH133-mediated tumor inhibition (Blázquez et al., 2003). These results were confirmed in glioma and astrocytoma xenografts, in which JWH133 inhibited VEGF, Ang1, Ang2, MMP-2, and TIMP-2 (Blázquez et al., 2003).

Cotreatment with the ceramide biosynthesis inhibitor Fumonisin B1 reversed the antitumor effect of JWH133, and its inhibitory effect on MMP-2 suggested that JWH133 downregulated MMP-2 expression underlying CB2R-mediated suppression of glioma cell invasion that occurred by inhibiting sphingolipid ceramide synthesis. These results were compared to findings with the mixed agonist Δ9-THC, suggested a critical role of CB2R in the Δ9-THC mediated effect.

The discovery of brain tumor stem cells has significant implications in developing new therapeutic approaches for managing malignant glioma and evaluating the benefits of currently available therapeutic medications (Maher et al., 2001). Aguado et al. (2007) reported that JWH133 stimulated glial cell differentiation in a CB2R-related manner, as shown by an increase in S-100β and GFAP and neuronal marker β-tubulin III in human glioma stem cells. Moreover, JWH133 reduced the cell population expressing the neuroepithelial progenitor marker nestin, causing a marked decrease in the efficiency of glioma formation *in vivo*, linked with reduced neurosphere formation and cell growth in secondary xenografts.

During parenchymal brain metastasis, cancer cells migrate through the brain endothelial cells that form the morphological basis of the blood–brain barrier (Wilhelm et al., 2013). Haskó et al. (2014) showed that CB2R stimulation by JWH133 decreased the adhesion of A2058 melanoma cells to the layer of hCMEC/D3 brain endothelial cells, indicating that CB2R activation on both endothelial and melanoma cells contributed to the adhesion-decreasing property of JWH133. JWH133 also reduced the rate of transmigration of melanoma cells, whereas coincubation with SR-144528 reversed these effects, verifying the CB2R-dependent effect of JWH133.

#### Lung Cancer

Non–small-cell lung cancer (NSCLC) is one of the common causes of cancer mortality worldwide. Despite this, only limited anticancer medications are available in current clinical practice (Jemal et al., 2009b). Preet et al. (2011) reported that JWH133 suppressed tumor growth and lung metastasis in SCID CB-17 mice inoculated with A549 cells. These antitumor effects were abolished by pretreatment with SR144528, indicating the direct involvement of CB2R in effect of JWH133. Moreover, JWH133 decreased tumor proliferation and neovascularization and enhanced apoptotic cell death in SCID CB-17 mice.

In another study, JWH133 exhibited cytotoxic activity in A549 cells and human umbilical vein endothelial cells (HUVECs) when used at the highest concentration (10^–4^ mol/L), whereas colony formation was prevented at non-toxic concentrations (10^−5^–10^−8^ mol/L) (Vidinský et al., 2012). Furthermore, JWH133 weakly induced DNA fragmentation in A549 cells. Furthermore, non-toxic concentrations of JWH133 inhibited some processes involved in angiogenesis and suppressed endothelial cell migration. JWH133 at 10^–4^ mol/L suppressed MMP-2 secretion. Thus, the antitumor activity of JWH133 occurred at micromolar concentrations in A549 cells.

Ramer et al. (2014) demonstrated that JWH133 in A549/huvec cocultures mitigated migration and tube and sprout formation in huvec. Inhibition was associated with the upregulation of tissue inhibitor of matrix metalloproteinases-1 (TIMP-1) and its upstream trigger ICAM-1, the intercellular adhesion molecule-1. The antiangiogenic effects of JWH133 are site-specific and limited to the tumor tissue. Indeed, conditioned media from JWH133-treated BEAS-2B cells, a normal bronchial epithelial cell line, did not prevent huvec migration. Therefore, JWH133 increased TIMP-1 production in lung cancer cells and induced ICAM-1 expression, thereby modifying the tumor cell microenvironment and inhibiting angiogenesis.

Considering the important role of macrophage-mediated vascular remodeling in several cancers, JWH133 significantly inhibited lipopolysaccharide-induced release of VEGF-A, VEGF-C, Ang1, and Ang2 and modestly affected IL-6 release in human lung macrophages (Staiano et al., 2016). However, JWH133 did not modulate the release of TNF-α or IL-8/CXCL8, and production of VEGF-A by human monocyte-derived macrophages was observed. CB2R activation by JWH133 inhibited the production of VEGF-A and VEGF-C from human lung macrophages but not from monocyte-derived macrophages. Stimulation of CB2R on tissue-derived macrophages could be a critical approach for the modulation of macrophage-mediated vascular remodeling in tumors and chronic inflammation.

#### Leukemia

Leukemias account for 30% of all pediatric cancers, and acute lymphoblastic leukemia (ALL) is the most prevalent pediatric leukemia, representing 75% of all pediatric leukemia cases (Terwilliger and Abdul-Hay, 2017). Punzo et al. (2018a) showed that JWH133 promoted apoptosis in patients with T-ALL and a Jurkat cell line via enhanced caspase-3 expression and Bax/Bcl-2 ratio. Moreover, JWH133 prevented tumor cell growth and survival via reduced expression of AKT, ERK, and Notch-1, while increasing the expression of PTEN and p53. This antitumor effect correlated with a remarkable inhibition of cell cycle progression by reducing the expression of cyclin-dependent kinase 2. Therefore, CB2R activation downregulated genes implicated in cell cycle progression and proliferation and upregulated genes implicated in apoptosis and cell cycle arrest in Jurkat cells.

#### Thyroid Carcinoma

Thyroid carcinoma is a malignant tumor of the endocrine system, which encompasses the majority of mortalities from endocrine tumors (Farid et al., 1994). Shi et al. (2008) reported that JWH133 induced a significantly greater apoptosis rate in ARO/IL-12 than in ARO thyroid carcinoma cells. Moreover, their findings were similar to those obtained when ARO cells were transfected with CB2 transgene (ARO/CB2). Intratumoral injection of JWH133 caused remission of thyroid tumors in nude mice inoculated with ARO/CB2 cells. CB2R was overexpressed after IL-12 expression in thyroid carcinoma cells. Thus, the upregulation of CB2R rendered thyroid cancer cells more responsive to CB2 agonist-induced apoptosis and led to tumor remission. Thus, the discovery of IL-12-mediated CB2 upregulation in thyroid tumors might provide a translational target for treating thyroid carcinoma.

#### Skin Cancer

The incidence of skin tumors has been increasing at a startling rate for several years. Various therapeutic agents have been identified, including cryotherapy, topical chemotherapeutic agents, and photodynamic therapy. However, these strategies have many limitations, including poor penetration of substances into the skin and difficulty accessing whole tumors (Leber et al., 1999). Casanova et al. (2003) found that incubating the tumorigenic mouse epidermal cell line PDV. C57 with JWH133 reduced cell viability by approximately 40%. JWH133 administration in nude mice inoculated with PDV. C57 cells caused approximately 60% reduction of tumor volume by inhibiting tumor vascularization as indicated by modified blood vessel morphology and downregulation of proangiogenic factors, including VEGF, placental growth factor, and angiopoietin-2, and inducing apoptosis. Activation of CB2R in tumor cells abolished EGFR function. It is possible that JWH133 interfered with the tumor angiogenic switch and directly stimulated tumor cell apoptosis, which in turn inhibited tumor proliferation. Therefore, both CB2R and EGFR might be critical for initiating signaling events that lead to tumor regression.

A similar study by Blázquez et al. (2006) demonstrated that JWH133 treatment resulted in tumor regression in nude mice bearing B16 melanoma cells, reducing tumor volume by approximately 75%, accompanied by an increase in apoptosis and reduced tumor vascularization and vascular density. Conversely, Luca et al. (2009) found that Kaposi sarcoma cells-treated JWH133 did not show remarkable inhibition of tumor proliferation and survival.

#### Osteosarcoma

Osteosarcoma (OS) is considered the most common bone cancer; it mainly affects children and teenagers and has a high rate of invasion and metastasis (Anderson, 2016). Punzo et al. (2017) reported that incubation with JWH133 induced apoptosis, upregulated caspase-3, and downregulated *p*-AKT in all OS cell lines studied (Saos-2, MG-63, MNNG/HOS, KHOS/NP, Hs888Lu, and U-2 OS). The antiproliferative activity of JWH133 was associated with the downregulation of Notch-1 and MMP-2, suggesting that JWH133 suppressed invasion/migration. Low-dose JWH133 decreased tumor growth and induced apoptosis, whereas higher doses had the opposite effect. Thus, CB2R stimulation exerted antiproliferative, proapoptotic, and antiinvasive effects; however, the dose should be considered while shifting to clinical setting.

In another study by the same group, activation of CB2R by JWH133 increased the efficacy of bortezomib in mediating apoptosis and decreasing invasion, arresting cell cycle progression, and modulating bone balance. Thus, they proposed that combining bortezomib with CB2R ligands in osteosarcoma therapy enables optimal dosing and reduces adverse effects (Punzo et al., 2018b).

### JWH133 in Cardioprotection

CB1 and CB2Rs are widely found in many tissues, including cardiac myocytes (Pertwee, 1997). The first indication that cannabinoids can be effective in ischemia was reported by Lagneux and Lamontagne (2001), who showed that cannabinoid receptors provided cardioprotection against lipopolysaccharide-triggered damage in isolated rat heart. Emerging evidence indicates that the CB2R acts during the early stages of ischemia–reperfusion, as shown by the decrease in infarct size in the presence of CB2 agonists before ischemia or during reperfusion in *ex vivo* preparations (Lépicier et al., 2006; Pacher and Haskó, 2008). The cannabinoid receptors have been involved in different cardiovascular disorders, including myocardial infarction, cardiomyopathy, arrhythmias, stroke, and cardiac arrest (Pacher et al., 2018).

#### Drug-induced Cardiotoxicity

Preincubation of myocardial HL-1 cells with JWH133 mitigated the histological alterations mediated by quetiapine (Li et al., 2019). JWH133 administration in mice resulted in a significant decrease in the ratio of heart weight to tibia length (HW/TL) and inhibited inflammatory cell infiltration and fibrosis. CB2R activation attenuated cell necroptosis by downregulating the expression of MLKL, phosphorylated MLKL, and attenuated RIP1 and RIP3. Thus, CB2R protected against quetiapine-induced cardiac toxicity by modulating necroptosis.

In another study by the same group, JWH133 reversed the elevated expression levels of p-RIP1, p-RIP3, and *p*-MLKL induced by ethanol in mice, indicating that CB2R may be the upstream signal molecules in necroapoptosis. Moreover, CB2R activation significantly ameliorated heart dysfunction, as indicated by increased left ventricular ejection fraction and fractional shortening and attenuated levels of cardiac injury markers (BNP, COL1Α1, TGF-β1, IL-1Β, and IL-6). The cardioprotective effect was associated with remarkable inhibition of inflammatory cell infiltration and fibrosis (Liu et al., 2020).

Pretreating mice with JWH133 suppressed clozapine-induced cardiotoxicity in mice, with a significant improvement in heart function and attenuation of infiltration index, fibrotic cardiac tissue, and serum cTnI levels (Li et al., 2019). Therefore, these findings proved the protective effects of CB2R activation against drug-induced cardiotoxicity.

#### Heart Failure

Myocardial hypertrophy is the increased myocyte mass elicited by hemodynamic stress or myocardial injury and is linked with a markedly increased risk of heart failure (Tanai and Frantz, 2015). Lu et al. (2014) found that low micromolar concentrations of JWH133 mitigated endothelin-1-elicited myocardial enlargement but did not attenuate endothelin-1-induced brain natriuretic peptide activation in isolated neonatal rat ventricular myocytes. Thus, CB2R stimulation might be a novel antihypertrophic cannabinoid therapy, which could improve the side effects of unopposed stimulation of CB1R alone.

The cardioprotective effects were validated by Maggo and Ashton (2018), who demonstrated that JWH133 did not influence atrial chronotropy in isolated rat atria, suggesting that CB2R activation did not induce tachycardia, whereas a CB1/CB2 agonist (WIN) and selective CB1 agonist (methanandamide) increased atrial chronotropy. Therefore, cannabinoid cardiotoxicity might include activation of CB1R in the heart, and CB2R agonists were not likely to have remarkable effects on the myocardium.

#### Myocardial Infarction

Acute myocardial infarction is the leading cause of death worldwide. Despite significant advances in restoring blood flow in the infarct area, reperfusion can damage the ischemic cardiac tissue (Yellon and Hausenloy, 2007). Montecucco et al. (2009) reported that JWH133 decreased the infarct size and severity of the cardiac injury, evidenced by reduced serum cTnI levels in mice. The cardioprotective effect of JWH133 was abrogated by pretreatment with AM630. JWH133 also attenuated ROS production and neutrophil infiltration in the infarcted myocardium, activated ERK1/2, which counteracted cardiac reperfusion injury, and enhanced STAT-3 expression. Pretreatment with the PI3K inhibitor LY294002, MEK1/2 inhibitor U0126, and JAK-2 inhibitor AG-490 partially blocked the JWH133-mediated mitigation of infarct size. JWH133 also inhibited human neutrophil migration in response to TNF-α by suppressing CD11b/CD18 (Mac-1) expression. Therefore, JWH133-mediated cardioprotection depends on the inhibition of oxidative stress and neutrophil recruitment and activation of the ERK 1/2 and STAT3 pathways.

JWH133 treatment significantly reduced the infarct size and apoptosis index of rat myocardium (Li et al., 2013). JWH133 maintained mitochondrial membrane potential (ΔΨm), downregulated the expression of caspases−3 and −9, inhibited the release of mitochondrial cytochrome c, and increased the expression of phosphorylated AKT. These effects were reversed by the PI3K inhibitors wortmannin and AM630. Thus, CB2R stimulation by JWH133 prevented apoptotic cell death during ischemia–reperfusion by suppressing intrinsic mitochondrial apoptosis via the PI3K/AKT signaling pathway.

In a similar study, JWH133 pretreatment remarkably improved ventricular function recovery during reperfusion, enhanced coronary flow, and decreased infarct size (Li et al., 2014). CB2R activation inhibited the loss of ΔΨm and mitochondrial permeability transition pore (MPTP) opening, decreased cytochrome c release into the cytosol, and upregulated *p*-ERK1/2 expression. These effects on the myocardium were abrogated by pretreatment with AM630 or the ERK1/2 inhibitor PD98059. Moreover, JWH133 counteracted atractyloside-induced MPTP opening. Thus, the cardioprotective effects of JWH133 during ischemia–reperfusion likely occur via phosphorylated ERK1/2 and preventing MPTP opening.

Defer et al. (2009) showed increased infarct size in CB2 knockout mice but reduced infarct size in wild-type mice treated with JWH133 at the time of reperfusion. Incubation with JWH133 protected cardiac myocytes from apoptosis induced by H_2_O_2_. However, the protective effect of JWH133 was diminished in CB2^−/−^ cardiac myocytes, and preincubation with AM630 confirmed the involvement of the CB2-dependent pathway. CBR2-mediated protection against apoptosis correlated with increased AKT phosphorylation and a reduced late apoptotic signal. Degradation of 45-kDa actin in cardiac myocytes suggested that CB2R stimulation increased cardiac myocyte resistance to oxidative damage by enhancing AKT signaling. In addition, JWH133 protected cardiac fibroblasts from H_2_O_2_-mediated apoptosis, limiting the release of TNF-α and α-SMA and inducing MMP-2 secretion. This protective effect was reversed in CB2^−/−^ fibroblasts. Therefore, CB2R activation provided cardioprotection by preventing oxidative stress-induced apoptosis in cardiac myocytes and fibroblasts and suppressing myofibroblast activation.

In another mouse model, JWH133 mitigated the severity of myocardial infarction by reducing infarct size, limiting myocardial enzyme expression (CK-MB and LDH), and improving cardiac function (Yu et al., 2019). Additionally, JWH133 protected primary cardiomyocytes as demonstrated by improved cell viability and LDH release. JWH133 attenuated the release of inflammatory cytokines (IL-1β, IL-18, IFN-γ, and TNF-α), and this effect was markedly reversed by AM630. JWH133 administration significantly inhibited the NLRP3 inflammasome in cardiac tissues of mice and in primary cardiomyocytes as indicated by the downregulation of NLRP3, casp1, and proIL-1β. Thus, the cardioprotective effect of CB2R activation relied on the modulation of NLRP3 inflammasome pathway.

### JWH133 in Metabolic Disorders

Metabolic syndrome is a complex pathological condition that involves several cardiovascular diseases, insulin resistance, and abdominal obesity (Kaur, 2014). Obesity is a potentially fatal metabolic disorder resulting from excessive calorie intake (Haase et al., 2014). Chronic inflammation associated with obesity is a core mechanism underlying obesity-related complications, including type 2 diabetes, non-alcoholic fatty liver disease, hypertension, atherosclerosis, and myocardial infarction (Van Gaal et al., 2006).

The cannabinoid system has a pivotal role in controlling energy metabolism (Engeli, 2012; Watkins and Kim, 2014). Several studies have demonstrated CB2R expression in peripheral metabolic tissues such as adipose tissue (Lin et al., 2017), the liver (Romero-Zerbo et al., 2012), pancreatic islet cells (Verty et al., 2015). Further, Ishiguro et al. (2010) found that Q63R, a common CB2R variant, causing decreased CB2 function, has been linked with eating disorders in humans. CB2 ligands reduce dietary intake in lean mice (Ishiguro et al., 2010) and ameliorate body weight and obesity-related inflammation in diet-induced obese mice (Verty et al., 2015). Moreover, CB2 genetic deficiency causes adiposity (Schmitz et al., 2016). This evidence suggests that CB2R ligands are a clinically viable therapeutic target for obesity.

Wu et al. (2020) examined the anti-inflammatory activities of CB2R and JWH133 in a diet-induced mouse model of obesity and cultured macrophages. They showed that JWH133 decreased body weight gain and adipocytic cell size, alleviated glucose intolerance, and enhanced insulin resistance. It also decreased the expression levels of M1 macrophage biomarkers (TNF-α, IL-6, iNOS, IL-1β, CCL2, and CXCL-10) while enhancing the expression of M2 macrophage biomarkers (IL-10 and arginase-1) in both mice and RAW264.7 macrophages. In both cases, the effects of JWH133 were blocked by pretreatment with AM630. JWH133 also inhibited the translocation of NF-κB p65 into the nucleus, enhanced the nuclear translocation of Nrf2, and upregulated the expression of HO-1 in cultured macrophages preincubated with LPS. However, the effect of JWH133 was reversed by an HO-1 inhibitor, Sn (IV) protoporphyrin IX dichloride. Thus, JWH133 exhibited antiobesity activity that attenuated proinflammatory M1 macrophage cytokines via Nrf2/HO-1.

In a clinical study, Rossi et al. (2016) found that the less-functional CB2-R63 variant was markedly correlated with a high z-score body mass index. Treatment of obese mouse-derived adipocytes with JWH133 showed decreased levels of PPARɣ, leptin, IL-6, and TNFα and increased expression of IL-4. The authors also observed a significant decrease in lipid droplet size and perilipin levels via CB2R-related modulation of PPARɣ. In addition, treatment of obese mouse-derived adipocytes with JWH133 resulted in significant upregulation of uncoupling protein-1 (UCP-1); this effect was abrogated by AM630 pretreatment. The evidence suggests that CB2R activation is a therapeutic target for mitigating obesity-associated inflammation and excess lipid storage in white adipose tissue by modulating perilipin expression, upregulating IL-4, and stimulating UCP-1 signaling.

In another study on the role of CB in controlling binge eating and obesity, it was reported that systemic administration of JWH133 produced a dose-dependent reduction in sucrose self-administration in wild-type and CB1^−/−^ mice, but not in CB2^−/−^ mice (Bi et al., 2020). However, pretreatment with AM251 accelerated and AM630 reversed the JWH133-mediated decrease in sucrose self-administration in wild-type mice, suggesting that cannabinoids inhibited this behavior by CB1R antagonism and CB2R agonism. Thus, JWH133 could decrease food rewarding and the motivation to seek sweetened food.

In contrast, Deveaux et al. (2009) reported that administration of JWH133 enhanced adipose tissue inflammation in HFD-fed mice. Moreover, exposure of cultured fat pads isolated from ob/ob mice to JWH133 showed increased expression of EMR1, TNF-α, and CCL2 (encoding MCP-1) in epididymal fat cells. Intraperitoneal administration of JWH133 enhanced HFD-induced insulin and hepatic steatosis in mice. These conflicting results suggest that CB2R activation mediates adipose tissue inflammation and enhances obesity-related insulin resistance and fatty liver.

#### JWH133 in Diabetes

Diabetes mellitus (DM), one of the most common metabolic diseases, is caused by a lack of insulin (T1DM) or reduced sensitivity and increased insulin resistance (T2DM) (Choi et al., 2015). DM often leads to numerous microvascular and macrovascular complications (Gruden et al., 2016).

Endocannabinoids modulate food consumption, glucose homeostasis, redox-inflammatory changes, and insulin release (Gruden et al., 2016). CB2Rs expressed in the islets of Langerhans mediate endocannabinoid signaling and endocrine secretion. CB2R stimulation increases insulin release from β-cells, inducing Ca^2+^ signalling (Juan-Picó et al., 2006). De Petrocellis et al. (2007) reported that treating rat insulinoma β-cells with JWH133 increased [Ca^2+^]i in the absence of extracellular Ca^2+^, whereas the inhibitor of phosphoinositide-specific phospholipase C (PI-PLC) U73122 resulted in a dose-dependent inhibition of intracellular Ca^2+^, which is the primary insulin release regulator in pancreatic β-cells. This observation may indicate that CB2R is coupled with enhanced [Ca^2+^]i via Gq/11-type G-proteins and stimulation of the phosphoinositide-specific phospholipase C cascade. Moreover, incubating rat insulinoma β-cells with JWH133 elevated [Ca^2+^]i independent of extracellular Ca^2+^, whereas preincubation with inhibitors of Ca^2+^ channels in the endoplasmic reticulum blocked the effect of JWH133. Thus, CB2R stimulation is associated with Ca^2+^ mobilization from endoplasmic reticulum stores.

In another study, McDonnell et al. (2017) found that JWH133 administration suppressed mechanical allodynia in db/db mice in a dose-dependent manner, whereas pretreatment with AM630 abrogated this effect. Stimulation of antioxidant Nrf2/HO-1 signaling by cobalt protoporphyrin IX (CoPP), a HO-1 inductor, and sulforaphane potentiated the antiallodynic effects of JWH133 and could be beneficial for the treatment of T2DM-associated neuropathic pain.

### JWH133 in the Reproductive and Hormonal System

#### Female Reproductive System

The endocannabinoid system is expressed in the female reproductive system of various species from sea urchins to humans, indicating its likely role in female reproduction (Sun and Dey, 2012). Components of the endocannabinoid system have been observed in the rodent and human uterus, and alterations in anandamide synthesis and expression of CB receptors in the uterus have been associated with early pregnancy failure or female infertility (Schmid et al., 1997). The expression and localization of cannabinoid receptors and enzymes in human oocytes and granulosa cells suggest that the endocannabinoid system plays a role in oocyte maturation (Agirregoitia et al., 2015).

Pagano et al. (2017) showed that JWH133 attenuated spontaneous uterine contraction induced by prostaglandin during the diestrus phase, whereas pretreatment with a CB2R blocker eliminated the spasmolytic effect of JWH133. JWH133 also reduced uterine contraction induced by exogenous PGE2 during the estrus phase, suggesting that the mechanism of action of JWH133 depends on the suppression of prostaglandin release and synthesis rather than on the selective effects on receptors present on smooth muscle. CB2R stimulation resulted in specific mitigation of myometrial contractility. These findings could be of interest to designers of tocolytic agents.

Ernst et al. (2016) found that CB2R activation by JWH133 significantly reduced basal but not FSH-activated estradiol and cytochrome P450 aromatase in the immortalized human granulosa cell line KGN. However, basal progesterone level and its FSH-induced stimulation remained unaffected after treatment with JWH133. Therefore, the intrinsic ovarian endocannabinoids showed modulatory effects in regulating estradiol synthesis.

#### Male Reproductive System

The cannabinoid system stimulates the mitotic–meiotic switch in male germ cells (Grimaldi et al., 2009). Active endocannabinoids have been reported in the testes and spermatozoa from mammals, sea urchins, and the frog *Rana esculenta* (Maccarrone et al., 2005; Schuel and Burkman, 2005). CB2R may also stimulate *in vitro* meiotic entry of postnatal male germ cells and sustain spermatogenesis progression *in vivo* (De Domenico et al., 2017). Thus, endocannabinoid agonism of CB2R may regulate meiotic entry and progression in germ cells.

Grimaldi et al. (2009) reported that CB2Rs were highly expressed throughout spermatogenesis with higher expression levels in spermatocytes (SPC). CB2R activation by JWH133 induced phosphorylation of ERK 1/2 MAPK in spermatogonia and their progression toward meiosis, as evidenced by an increase of synaptonemal complex protein (SCP3), a marker of meiotic prophase, and upregulation of early meiotic prophase genes (c-Kit, Dmc1, and Stra8). However, this effect was abrogated by pretreatment with AM630, indicating a prodifferentiation function of CB2Rs in male germ cells. A similar study by Di Giacomo et al. (2016) demonstrated that JWH133 stimulated the expression of the meiotic genes c-Kit and Stra8 through upregulation of H3K4me3 and downregulation of H3K9me2 in isolated spermatogonia (SPG). Moreover, JWH133 upregulated the Prdm9 gene, which encodes a meiosis-specific histone, H3K4me3 methyltransferase. Chronic administration of JWH133 to immature 7 dpp CD-1 mice accelerated spermatogenesis, whereas CB2 blockade retarded it, suggesting that CB2R hyper- and hypoactivation disrupted the progression of the spermatogenic cycle. The contribution of CB2Rs to the physiological control of spermatogenesis might provide novel therapeutic strategies for treating infertility in humans.

De Domenico et al. (2017) reported that CB2R activation by JWH133 triggered meiosis by elevating SCP3 populations, including preleptotene and leptotene spermatocytes but not in more advanced stages, and indicated that CB2R stimulation facilitated entry and progression of the early stages of meiosis in fetal and postnatal male germ cells. However, they did not repress meiotic checkpoints to move toward the end of prophase I. Additionally, JWH133 upregulated the expression of the meiotic genes Stra8, Kit, Scp1, Scp3, and Dmc1 and downregulated Nanos2—these effects were reversed by pretreatment with AM630. The effect of JWH133 was accompanied by induction of apoptosis, indicating that meiosis facilitation by JWH133 was not followed by DNA repair, thus enhancing oocyte apoptotic rate. Interestingly, JWH133 treatment of pregnant females from E12.5 to E16.5 attenuated primordial and primary follicles in ovaries of newborns with subsequent exhaustion of ovarian store and decreased fertility in adulthood, without affecting spermatogenesis in the offspring’ testis. These results highlight the promeiotic function of CB2R in male and female germ cells and suggest that the use of cannabis during pregnancy is a risk for fertility and reproduction in female offspring.

In contrast, Innocenzi et al. (2019) reported that prolonged treatment of P7 CD-1 male mice with JWH133 reduced sperm count, inhibited placental development, and decreased offspring growth, suggesting an overall negative effect on embryo growth. These deformities were accompanied by modified DNA methylation/hydroxymethylation at imprinted genes in sperm that was preserved in the placenta. Thus, overactivated CB2Rs altered sperm DNA methylation patterns that might be inherited and induced negative consequences on offspring growth, underlining the possible risks of recreational use of cannabinoids.

### JWH133 in Gastrointestinal Disorders

The gastrointestinal endocannabinoid system is implicated in regulating motility, sensation, and intestinal inflammation (Unal et al., 2020). CBR2 is found throughout the GI tract, with expression dependent on the presence of inflammation (Ambrose and Simmons, 2019). Moreover, CB2Rs have been identified on enteric neurons, where they are implicated in the regulation of intestinal motility during inflammation (Duncan et al., 2008).

#### Inflammatory Bowel Disease

Crohn’s disease and ulcerative colitis are chronic intestinal inflammatory pathologies collectively known as inflammatory bowel disease (IBD), which is considered a significant health problem currently (Loftus, 2004). Storr et al. (2009) reported that JWH133 treatment mitigated trinitrobenzene sulfonic acid-induced colitis in mice was associated with a remarkable attenuation of inflammation, as demonstrated by reduced macroscopic damage score, colonic adhesions, and myeloperoxidase activity. However, cotreatment with AM630 and JWH133 abrogated the protective effects of JWH133, indicating CBR dependence.

Furthermore, Kimball et al. (2006) found that prophylactic low-dose of JWH133 mitigated colon weight gain, colon shrinkage, macroscopic inflammatory damage score, diarrhea, and pathological damage in a mustard oil-induced model of colitis in CD-1 mice. JWH133 ameliorated microscopic and macroscopic inflammatory damage scores when administered in a prophylactic dose to mice with dextran sulfate sodium-induced colitis, although relatively higher doses of 10 or 20 mg/kg were needed, indicating that JWH133 was less efficient than the CB1R agonist ACEA.

In another study by Singh et al. (2012), JWH133 mitigated colitis-related pathogenesis and decreased body weight in IL-10^−/−^ mice. This was accompanied by a significant decrease in the percentage of CD4^+^ T cells, neutrophils, mast cells, natural killer cells, and activated T cells in the intestinal lamina propria. Thus, JWH133 abrogated colitis through inhibition of Th cell stimulation by facilitating apoptotic cell death, thereby reducing the production of other inflammatory cells at inflamed sites in the colon. In addition, JWH133 improved dextran sodium sulfate-induced colitis, indicated by a significant reduction in macrophage number and percentage and IFN-γ expression. JWH133 administration stimulated T-cell apoptosis *in vivo* and *in vitro*, whereas AM630 abrogated the protection mediated by JWH133. Cumulatively, CB2R activation by JWH133 mediated anti-inflammatory activities by inhibiting T-cell activation and inducing apoptosis.

In a clinical study, mucosal samples were obtained from the inflamed/uninflamed colon of patients with IBD and Caco-2 cells (Tartakover Matalon et al., 2020). JWH133 did not influence epithelial apoptosis but augmented epithelial/stromal cell proliferation, indicating that enhanced epithelial cell growth could result from the direct action of JWH133 on the epithelial cells or because of a cross-link between CB2R-expressing stromal cells and epithelial cells. Moreover, CB2R stimulation decreased secretome MMP9 and IL-8 levels in inflamed areas. Secretomes of JWH133-treated biopsies showed enhanced Caco-2 number, migration, proliferating cell nuclear antigen, and autophagic LC3IIB expression but did not affect permeability. Therefore, CB2R activation might stimulate mucosal healing in patients with IBD.

#### Gastrointestinal Motility

Kimball et al. (2010) reported that JWH133 exerted dose-dependent attenuation of small intestinal transit in mustard oil-induced colitis in mice. A dose of 1 mg/kg JWH133, alone or in combination with a CB1R-specific agonist, ACEA, significantly decreased the small intestinal transit in colitis mice compared with that in control mice. CB2R was highly expressed in the lamina propria on day 28 after colitis induction. Therefore, CB2R remodeling occurred during GI inflammation and continued throughout the recovery phase, resulting in increased JWH133 efficacy. Thus, CB2R-specific agonists might improve GI motility in patients suffering from diarrhea-predominant IBS. However, a study by Baldassano et al. (2008) showed that JWH133 at 0.1–10 μM did not cause a dose-dependent decrease in spontaneous contraction in mouse ileal longitudinal muscle; therefore, it did not modulate intestinal motility. Indeed, CB2R in the rat intestine has contributed to GI transit mitigation only following inflammatory stimulus (Mathison et al., 2004). In mice, CB2R function depends on the region of the digestive tract in which it is expressed. CB2R stimulation is ineffective in the colon (Mulè et al., 2007); however, its activation by JWH133 attenuates cholinergic contraction in the stomach, an effect that is reversed by AM630 (Mulè et al., 2007).

Mathison et al. (2004) showed that JWH133 did not affect basal transit but suppressed LPS-mediated GI transit, which was reversed by AM630. JWH133 seemingly acted via cyclooxygenase and independent of iNOS and platelet-activating factor. Thus, CB2R stimulation in response to LPS reestablished regular GI transit following inflammation. This observation was confirmed by Li et al. (2010), who revealed that JWH133 decreased myoelectrical activity, whereas AM630 did not, indicating that CB2Rs do not modulate myoelectrical activity under normal conditions. They also noted that CB2 agonists did not affect upper GI transit under basal conditions.

Similarly, Duncan et al. (2008) identified CB2Rs on enteric neurons. JWH133 did not influence the twitch response of electrically stimulated ileum under physiological conditions but exerted a dose-dependent reduction in LPS-accelerated contraction in rats. Further, JWH133 downregulated the Fos expression induced by LPS in both enteric glia and neurons. This action was blocked by AM630; thus, CB2R stimulation in the enteric neurons of the GI tract decreased the endotoxin-induced accelerated intestinal contractility.

#### Pancreatitis

Michler et al. (2013) found that JWH133 ameliorated cerulein-induced acute pancreatitis, thereby reducing trypsin activity in pancreatic tissue, myeloperoxidase activity in lung tissue, and IL-6 levels in serum as well as mitigating histological alternations. This was accompanied by inhibition of intra-acinar JNK stimulation and suppression of apoptosis. Pretreatment with JWH133 enhanced p38 phosphorylation in both wild-type and MK2^−/−^ mice. However, the protective effects of JWH133 were reversed after pretreatment with AM630 or in MK2 knockout mice, validating the dependence of JWH133 on CB2R.

Suppression of JNK and stimulation of p38 as well as the MK2-signaling pathways may be responsible for mediating the beneficial effects of CB2R stimulation during acute pancreatitis. Moreover, Xia et al. (2019) showed that JWH133 prevented acetylcholine-induced Ca^2+^ oscillations in mouse pancreatic acinar cells, whereas CB2R-knockout or AM630 blocked the suppressive effects of JWH133. Thus, CB2R activation might play a novel role in modulating the physiology and pathophysiology of pancreatic acinar cells.

### JWH133 in Hepatic Diseases

**FIGURE 4:**
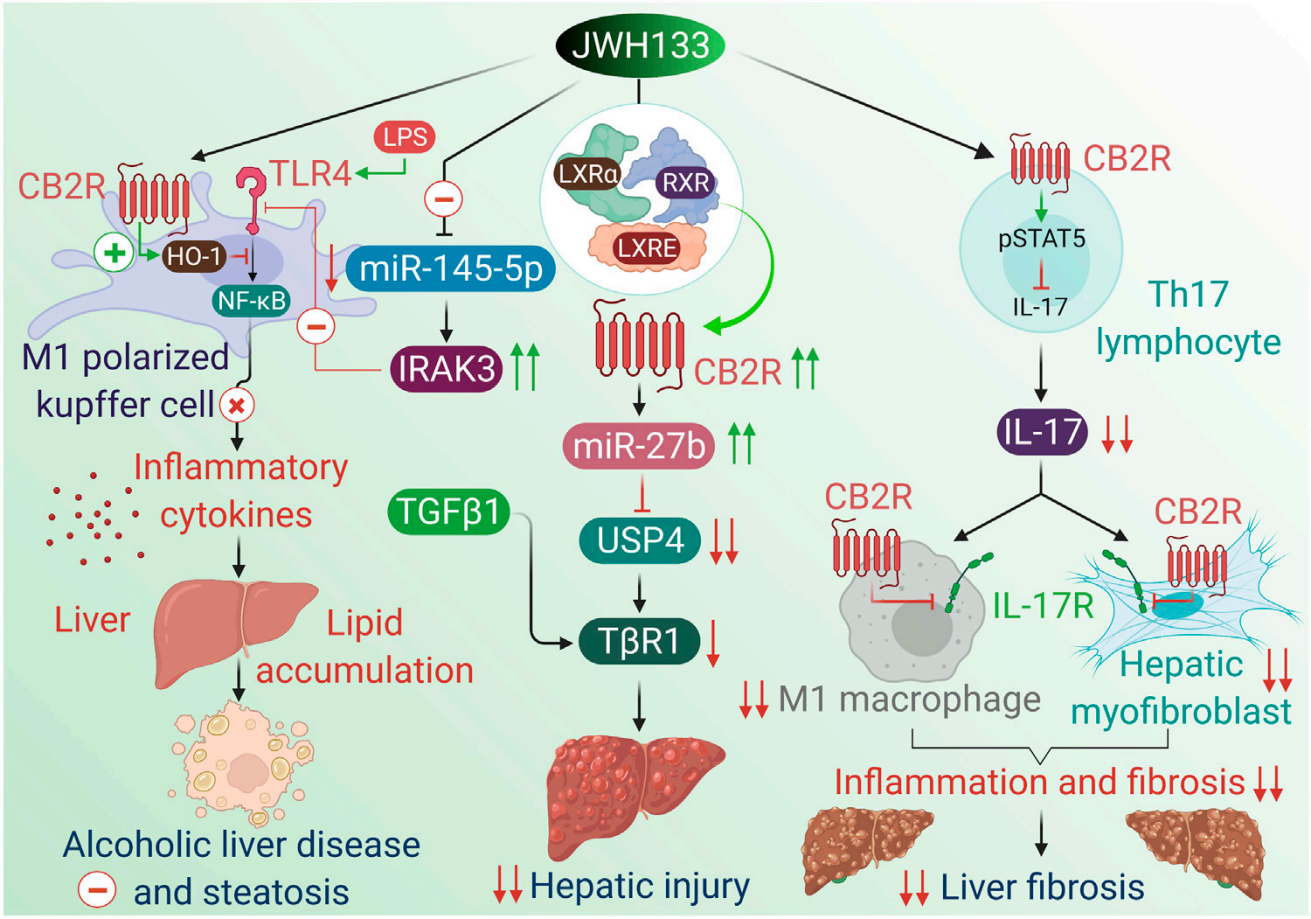
The hepatoprotective effects and mechanism of JWH133.

Endocannabinoids are expressed at lower levels in the liver under normal basal conditions and are markedly increased after hepatocyte injury (Caraceni et al., 2010). CB2R stimulation has anti-inflammatory and antifibrogenic activities. It mitigates paracetamol-induced liver injury (Rivera et al., 2020), cirrhosis (Dibba et al., 2018), non-alcoholic fatty liver disease (Mendez-Sanchez et al., 2007), and alcoholic liver disease (Louvet et al., 2011) in experimental models. Thus, targeting the cannabinoid system might attenuate liver injury and reduce the incidence of complications. The hepatoprotective effects and mechanisms of JWH133 are presented in Figure 4.

#### Acute Liver Failure

Acute liver injury (ALI) is characterized by sudden onset of severe dysfunctional hepatocytes and has been correlated with viral hepatitis, drug toxicity, exposure to toxins, and unknown reasons (Zhan et al., 2014). Tomar et al. (2015) reported that JWH133 attenuated GalN/LPS-induced elevation of mortality rate; release of alanine transaminase and inflammatory cytokines (TNF-a, MCP-1, and IL-6), histological alterations, hepatic apoptotic damage, and liver infiltration of mononuclear cells in ALI mice. These effects were accompanied by a significant increase in the production of anti-inflammatory cytokine IL-10 in M1 macrophages, and upregulation of M2 markers (Arg-1 and Chi3L3) in M2 macrophages suggested that JWH133 suppressed M1 stimulation while potentiating the M2 phenotype. Similarly, JWH133 treatment of ALI mice inhibited ALF-mediated expression of M1 markers (TNF-α and IL-12) while upregulating M2 markers (Arg1, IL-10) in liver mononuclear cells. JWH133 downregulated miR-145 expression, which in turn led to a significant upregulation of interleukin-1 receptor-associated kinase 3 (IRAK3), a negative regulator of TLR4 signaling. Cumulatively, CB2 activation could mitigate GalN/LPS-induced ALF by mediating the M1 to M2 transition in macrophages and modulating miR-145 expression to hamper TLR4 signaling following LPS-triggered inflammation.

Killilea et al. (2020) showed that pretreatment with low-dose JWH133 did not attenuate LPS/GalN-induced ALI in Sprague–Dawley or WKY rats at 6 h. These results indicated a lack of CB receptor-mediated protection in ALI SD or WKY rats, and protective effects could be noted with higher doses of JWH133 over different time intervals (e.g., 24 h) after prolonged administration. Further studies are needed to determine whether CB2R activation stimulates or mitigates severe liver injury in stress-sensitive rats.

#### Alcoholic Liver Disease

Alcoholic liver disease (ALD), a principal cause of morbidity and mortality globally, involves a broad spectrum of diseases, ranging from the relatively benign fatty liver to more severe liver injury (Gao and Bataller, 2011).

Louvet et al. (2011) demonstrated that treatment of alcohol-fed mice with JWH133 mitigated hepatic M1 gene expression (TNF-α and the chemokines CCL3, CCL4, and IL-6) without influencing M2 macrophages, indicating that endogenous or exogenous stimulation of CB2R suppressed alcohol-mediated M1 polarization of Kupffer cells. Further, CB2R activation by JWH133 led to significant modulation of alcohol-induced fatty liver, as demonstrated by the attenuation of liver steatosis in mice and its acceleration in CB2^−/−^ mice. Additionally, JWH133 inhibited M1 polarization and mediated the shift to M2 macrophages in isolated Kupffer cells and cultured macrophages, thereby protecting against lipid accumulation in hepatocytes via paracrine effects. In cultured macrophages and alcohol-fed mice, JWH133 also upregulated the expression of heme oxygenase-1, whereas the HO-1 inhibitor zinc protoporphyrin blocked the preventive effect of JWH133 on LPS-induced NF-κB stimulation and M1 polarization, indicating that CB2R activation affords anti-inflammatory effects by upregulating HO-1 in macrophages.

Furthermore, Denaës et al. (2016) showed that CB2R stimulation by JWH133 augmented autophagy, as evidenced by increased accumulation of LC3-II and reduced SQSTM1/p62 levels via HO-1 pathway in cultured RAW264.7 macrophages. Moreover, JWH133 mitigated the release of LPS-induced proinflammatory genes (CCL4, IL-1, CCL3, and IL-6, iNOS) in cultured macrophages but not in ATG5-deficient cells. Confirming these results *in vivo*, they found that JWH133 protected wild-type mice from alcohol-induced hepatic inflammation and steatosis; however, this was not noted in ATG5Mye^−/−^ mice, demonstrating that autophagic process in macrophages mediate the anti-inflammatory and antisteatogenic activities of CB2Rs.

#### Liver Cirrhosis

Muñoz-Luque et al. (2008) reported that JWH133 ameliorated arterial pressure, reduced the infiltration of inflammatory CD68 cells, and reduced activated stellate cells while enhancing apoptotic cell death in myofibroblastic and monocytic cells and reducing fibrosis in cirrhotic rats with ascites induced by CCl4. The authors also observed reduced α-SMA and collagen I and enhanced MMP-2 expression in cirrhotic rat liver. Therefore, selective stimulation of hepatic CB2R caused a significant decrease in hepatic collagen levels in cirrhotic rats, indicating that selective CB2 agonists might be a therapeutic agent for liver fibrosis.

Huang et al. (2012) found that JWH133 reduced mesenteric vascular density, mesenteric angiogenesis, and portosystemic shunting in cirrhotic rats induced by bile duct ligation. Because mesenteric blood flow is the major contributor of portal blood inflow, its suppression by JWH133 mitigates liver fibrosis. Yang et al. (2014) reported that prolonged JWH133 treatment alleviated portal hypertension, systemic/intestinal oxidative damage, associated inflammation, infection, intestinal mucosal damage, and hyperpermeability in cirrhotic ascitic rats. The authors observed a significant reduction in bacterial overgrowth and adhesion; decrease in spontaneous bacterial peritonitis; upregulation of intestinal tight junction genes, namely, occludin, claudin, and ZO-1; and downregulation of TNF-α-receptor/NF-kBp65 protein expression in peritoneal macrophages. Additionally, acute and chronic JWH133 treatment protected against the TNFα-mediated inhibition of phagocytosis of peritoneal macrophages in cirrhotic rats, an effect that was abrogated by cotreatment with AM630, suggesting that chronic CB2R stimulation by JWH133 markedly improved the phagocytosis of peritoneal macrophages in cirrhotic rats by suppressing TNF-α signaling, proinflammatory cytokine secretion, and oxidative stress. Therefore, CB2R ligands might be beneficial for treating bacterial translocation in cirrhosis.

Steib et al. (2013) found that pretreatment with JWH133 mitigated portal hypertension following Kupffer cell activation in cirrhotic rats induced by BDL. Further, JWH133 upregulated the expression of HO-1, whereas treatment with the HO-1 inhibitor ZnPP IX accelerated portal hypertension, indicating the beneficial role of HO-1 signaling. In isolated Kupffer cells activated by either Zymosan or LPS, JWH133 treatment significantly increased the expression of CB2 and HO-1, while reducing the expression of the vasoconstrictor TXB2. HO-1 reduces portal pressure via its anti-inflammatory activity (Angermayr et al., 2006), leading to decreased TXB2 production. Pretreatment with the PPARγ inhibitor GW9662 blocked JWH133-induced attenuation of portal hypertension and upregulation of HO-1. Therefore, PPARγ might be the link between CB2R and HO-1. CB2R activation mediating the HO-1 pathway could be a beneficial target for patients with liver cirrhosis-associated portal hypertension.

#### Liver Fibrosis

Teixeira-Clerc et al. (2010) reported that CB2R activation by JWH133 led to a significant reduction in liver apoptosis and acceleration of hepatic regeneration measured by the increased onset of PCNA induction in CCl4-treated mice. Incubating hepatic myofibroblasts with JWH133 enhanced the expression of TNF-α and IL-6 and reduced the expression of MMP-2 as myofibroblasts secrete bioactive cytokines with antiapoptotic and mitogenic effects, such as TNF-α and IL-6 (Lotersztajn et al., 2005). Thus, CB2R mitigated hepatic injury and promoted regeneration through a paracrine mechanism, including hepatic myofibroblasts, suggesting that CB2 ligands demonstrate hepatoprotective activities as well as antifibrogenic effects.

Guillot et al. (2014) showed that incubation of T-helper (Th17) lymphocytes with JWH133 reduced the differentiation of CD41-naive T cells into Th17 lymphocytes and was accompanied by decreased Th17 marker expression and IL-17 secretion. IL-17 is a proinflammatory and fibrogenic cytokine mainly produced by Th17 lymphocytes. It did not alter the release of antifibrogenic IL-22. However, the suppressive effects of JWH133 were abrogated in Th17 lymphocytes obtained from CB2-knockout mice. Further, JWH133 increased the phosphorylation and translocation of STAT5 into the nucleus, a function that was blocked by adding a STAT5 inhibitor. Finally, CB2R stimulation in macrophages and hepatic myofibroblasts showed blunted IL-17-induced expression of proinflammatory genes. Cumulatively, CB2R stimulation decreased liver fibrosis by specifically decreasing IL-17 production by Th17 lymphocytes in a STAT5-dependent manner and decreasing the proinflammatory activity of IL-17 while conserving IL-22 production.

Wu et al. (2019) found that treating mice with JWH133 and CCL4 plus clodronate inhibited toxicant-mediated hepatic injury, as demonstrated by reduced levels of ALT, AST, apoptotic cells, caspase-3, and CREB. JWH133 also attenuated protein kinase A activity except in CB2-deficient mice, demonstrating that hepatocytic cells express functionally active CB2 downstream of the liver X receptors (LXRα). Additionally, JWH133 administration suppressed TGF-β1-mediated cleavage of caspase-3 in AML12 cells and reduced ubiquitin-specific peptidase 4 (USP4), indicating that LXRα stimulation of CB2 destabilized TGF-β receptor 1 (TβRI), an upstream sensing molecule via USP4 suppression. This result was associated with significant upregulation of miR-27b, an inhibitor of USP4. Thus, LXRα could exert a protective effect against TGF-β by transcriptional regulation of the CB2R gene in hepatocytes, and then CB2 might inhibit USP4-stabilizing TβRI via miR-27b.

#### Hepatic Ischemia-Reperfusion

Bátkai et al. (2007) reported that CB2R activation by JWH133 markedly decreased transaminase levels, attenuated oxidative stress, and reduced the infiltration of inflammatory cells, as demonstrated by reduced levels of MPO activity, TNF-α, MIP-1α, MIP-2, and ICAM-1 following ischemia–reperfusion in mice. Furthermore, JWH133 mitigated TNF-α-stimulated ICAM-1 and VCAM-1 expression in human liver sinusoidal endothelial cells (HLSECs) and decreased the adhesion of human neutrophils to HLSECs. However, this protective effect was completely abolished by cotreatment with SR144528 or in CB2^−/−^ mice, indicating the dependence on CB2R.

Reifart et al. (2015) found that JWH133 pretreatment downregulated α-SMA in I/R mice, and hepatic stellate cell activity was negatively affected by CB2R activation. HSC deactivated by JWH133 exhibited markedly reduced CD4^+^ T-cell migration in the postischemic liver. JWH133 resulted in significant improvement of postischemic perfusion and decreased liver injury. Thus, the deactivation of hepatic stellate cells by JWH133 attenuated CD4^+^ T-cell recruitment and decreased microvascular and hepatocellular injuries. Thus, hepatic stellate cells could be a clinical target for novel therapeutic approaches for T-cell-induced I/R injury during liver transplantation.

### JWH133 in Autoimmune Disorders

CB2Rs are expressed by all immune cells with varying expression between immune cells and activation conditions (Suárez-Pinilla et al., 2014). CB2Rs from hematopoietic cells promote cannabinoid-induced immune modulation (Munro et al., 1993). Synthetic CB2R agonists significantly suppressed autoimmunity in different animal models, including collagen-induced arthritis (Malfait et al., 2000), experimental autoimmune encephalomyelitis (Sánchez et al., 2006), and virus-mediated demyelinating disease (Arévalo-Martín et al., 2003).

### Rheumatoid Arthritis

Rheumatoid arthritis (RA) is a chronic autoimmune disorder characterized by prolonged inflammation of the synovium, resulting in bone and cartilage destruction (McInnes and Schett, 2017). Fukuda et al. (2014) found that JWH133 prevented the secretion of IL-6, MMP-3, and CCL2 from TNF-α-activated fibroblast-like synoviocytes obtained from the rheumatoid joints. Further, coincubating peripheral blood CD14^+^ monocytes with JWH133 caused a dose-related suppression of osteoclast formation and inhibition of M-CSF and RANKL-mediated calcium resorption. Additionally, JWH133 treatment of mice with collagen-induced arthritis (CIA) decreased the arthritis score and reduced inflammatory cells’ infiltration, bone destruction, and antiCII IgG1 release. Thus, CB2R activation might be a beneficial target for RA by inhibiting the production of proinflammatory cytokines from fibroblast-like synoviocytes and preventing the formation of bone-resorbing cells.

Zhu et al. (2019) showed that JWH133 mitigated synovial hyperplasia, associated inflammation, cartilage damage, and bone destruction in CIA mice, indicating the remarkable protective activity of JWH133 against arthritis and local bone loss in the CIA mice. JWH133 injection decreased the infiltration of proinflammatory M1-like macrophages and promoted macrophage repolarization from the M1 to M2 phenotype. The authors also observed upregulation of the anti-inflammatory cytokine IL-10 and downregulation of inflammatory mediators, such as TNF-a, IL-1b, and IL-6. Moreover, JWH133 treatment alleviated osteoclast formation and bone resorption and downregulated the expression of RANKL-induced NF-kB activation, MMP-9, tartrate-resistant acid phosphatase, cathepsin K, and nuclear factor of activated T cells 1 (NFAT-1) in CIA mice and osteoclast precursors. These effects were abolished by cotreatment with SR144528. JWH133 also downregulated the expression of p65-positive cells in CIA mice. Thus, JWH133 inhibited osteoclastogenesis and inflammation-mediated bone destruction by inhibiting NF-kB signaling, thereby highlighting its clinical potential as a therapeutic agent for human RA.

Conversely, Fechtner et al. (2019) reported that pretreatment of RA synovial fibroblasts (RASFs) with JWH133 did not attenuate IL-1β-mediated IL-6 and IL-8 production and upregulated the expression of COX-2. However, these effects were reversed in CB2-deficient mice. Further, MMP-2 and MMP-9 activities were decreased in CB2-deficient mice. In contrast, activation of CB2 in RASFs augmented the IL-1β-induced IL-6, IL-8, RANTES, and ENA-78. They also found that JWH133 coordinated the CB2R association with TGFβ-activated kinase 1, a key signaling molecule, increasing the IL-1β-induced nuclear translocation of NF-κBp65 and activation protein-1. This conflicting data showed that pharmacological activation of CB2R mediated IL-1β-induced inflammation in RASFs, whereas genetic deletion of CB2R in mice alleviated the inflammation induced by IL-1β, thus highlighting the role of the CB2R in managing RA pain and inflammation.

#### Immune Thrombocytopenia

Immune thrombocytopenia (ITP) is a complex autoimmune disease marked by antibody-stimulated platelet destruction (Khan et al., 2017). Rossi et al. (2019a) reported that incubating ITP-mesenchymal stromal cells with JWH133 and Dexa, alone or in combination, significantly reduced the expression of the inflammatory mediator IL-6 and induced the expression of IL-4. These effects were reversed by AM630, thereby verifying the dependence of these effects on CB2R. CB2R activation by JWH133 and Dexa attenuated apoptosis by stimulating Bcl2 signaling and restored mesenchymal stromal immunomodulation. This effect was blocked by AM630, indicating the dependence of mesenchymal stromal cell immunosuppression on CB2R. These findings suggest that the combination of Dexa with JWH133 is beneficial in ITP, decreasing the dose requirements and incidence of adverse effects.

#### Autoimmune Uveoretinitis

Experimental autoimmune uveoretinitis (EAU) in rats and mice is a prototypic T-cell-induced autoimmune disorder targeting the neural retina and associated tissues (Caspi, 2003). Xu et al. (2007) showed that JWH133 inhibited EAU in mice by suppressing disease induction and effector stages. JWH133 also abolished cytokine/chemokine production (TNF-α, IL-6, IL-10, INF-γ, CCL2). Additionally, treating EAU mice with JWH133 inhibited leukocyte trafficking in the inflamed retina because of its effect on attenuating adhesion molecules CD162 (P-selectin glycoprotein ligand 1) and CD11a (LFA-1) expression on T cells. Leukocytes isolated from JWH133-treated mice exhibited a reduced response to activation by retinal peptide and mitogen Concanavalin A. Downregulation of TLR4 via Myd88 signaling may be responsible for the inhibitory effects on antigen presentation. Taken together, CB2R activation by JWH133 produced an anti-inflammatory effect by suppressing the stimulation and function of autoreactive T cells and averting leukocyte trafficking into the inflamed retina.

#### Systemic Sclerosis

Systemic sclerosis (SSc) is an autoimmune connective tissue disease marked by inflammation and intensive fibrosis of the skin and visceral organs (LeRoy and Medsger, 2001). Servettaz et al. (2010) found that CB2R activation by JWH133 suppressed the development of skin fibrosis, with significantly reduced dermal thickness and collagen content in the skin and lungs of hypochlorite-induced SSc mice. JWH133 also reduced pulmonary T-cell infiltration and counteracted the increase in splenic B cell numbers, decrease in fibroblast growth, and the development of autoantibodies (antiDNA topoisomerase1 IgG Abs). However, these effects were blocked in CB2R-knockout mice, confirming the impact of CB2R in systemic fibrosis and autoimmunity.

### JWH133 in Renal Disorders

CB2Rs are expressed in glomeruli and tubules in human and rat kidneys (Jenkin et al., 2010; Barutta et al., 2011). CB2R also localizes to the bladder tissue of different species, such as humans, rodents, and monkeys (Gratzke et al., 2010; Li et al., 2013). Earlier studies have revealed that the levels of endocannabinoids and CB2Rs in renal ischemia are linked with renal damage (Moradi et al., 2016; Pressly et al., 2018). CB2 stimulation reduced renal damage and CB2 antagonism increased kidney damage in various experimental models of nephropathy (Jenkin et al., 2016; Zoja et al., 2016).

#### Renal Ischemia-Reperfusion Injury

Kidney IR injury is a pathological condition that leads to acute kidney failure (Hsu et al., 2007). Feizi et al. (2008) reported that JWH133 administration resulted in dose-dependent inhibition of reperfusion-induced ischemia-mediated lesions in mouse kidneys. These results were confirmed by Çakır et al. (2019a), who found that treatment with three different doses of JWH133 significantly mitigated the glomerular and tubular injury in rats. This was accompanied with a significant reduction in the levels of renal NF-κB, TNF-α, IL-1β, and caspase-3.

Likewise, JWH133-treated rats showed a remarkable decrease in the serum levels of TNF-alpha, blood urea nitrogen, creatinine, kidney injury molecule-1, neutrophil gelatinase-associated lipocalin, cystatin C, IL-18, IL-1β, IL-6, and IL-10. Therefore, CB2R activation by JWH133 ameliorated pathological kidney damage by suppressing inflammatory cytokine secretion and apoptosis. JWH133 could be a novel therapeutic agent in the prevention of renal IR injury.

#### Interstitial Cystitis/Bladder Pain Syndrome

Furthermore, Liu et al. (2020a) showed that JWH133 diminished mechanical hyperalgesia, reduced urine spot numbers, and enhanced the micturition frequency mediated by cyclophosphamide-induced cystitis in mice. They also observed a reduction in bladder tissue inflammation and oxidative damage as indicated by reduced levels of proinflammatory mediators, including IL-1β, TNF-α, and IL-8, and enhanced activities of cellular GSH and SOD, while lowering MDA levels. CB2R stimulation by JWH133 induced autophagy via upregulation of LC3-II/LC3-I and downregulation of SQSTM1/p62 in mouse bladder tissue. However, treatment with AM630 abolished these protective effects. Cotreatment with the autophagy inhibitor 3-methyladenine also blocked the influence of JWH133 on inflammation and oxidative injury. Furthermore, JWH133 upregulated *p*-AMPK expression and downregulated *p*-mTOR expression, whereas pretreatment with 3-methyladenine blocked this effect. Thus, CB2 stimulation in the bladder mitigated the severity of cyclophosphamide-induced cystitis and improved bladder inflammatory responses by activating autophagy and AMPK-mTOR signaling.

### JWH133 in Skin Diseases

CB2Rs are localized in the skin, indicating that CB2 signaling could have a role in dermal fibrosis (Blázquez et al., 2006; Karsak et al., 2007). Akhmetshina et al. (2009) reported that JWH133 mitigated the profibrotic activity of bleomycin and reduced dermal thickening in bleomycin-induced fibrosis in mice. CB2 mediated its antifibrotic effects in mice by preventing leukocyte infiltration into skin lesions in mice treated with JWH133. Thus, CB2 signaling could indirectly influences dermal fibrosis by modulating leukocyte infiltration rather than direct action on the collagen synthesis in fibroblasts. However, preventing CB2 signaling by gene inactivation or CB2R blockade enhanced the vulnerability to bleomycin-induced dermal fibrosis. These findings suggest CB2 activation is a promising strategy for treating the early inflammatory stages of systemic sclerosis. Norooznezhad and Norooznezhad (2017) suggested using oral or topical JWH133 for psoriasis owing to its ability to inhibit keratinocyte proliferation and prevent angiogenesis and inflammation. However, further *in vivo* studies and clinical trials are needed.

### JWH133 in Respiratory and Lung Diseases

CB receptors in rat and human pulmonary artery endothelial cells can be stimulated to reduce oxidative damage and inflammation (Luchicchi and Pistis, 2012). Previous studies have demonstrated that smoking marijuana or ingestion of 9-tetrahydrocannabinol (THC) results in bronchodilation (Tashkin et al., 1974; Tashkin et al., 1975). In the lung tissue, activation of CB1 or CB2Rs can suppress C-fiber-induced responses, such as neurogenic inflammation, bronchoconstriction, and cough (Patel et al., 2003; Fukuda et al., 2010). Thus, treatments targeting CB receptors could help manage airway hyperresponsiveness and asthma (Pini et al., 2012).

#### Lung Injury

Paraquat (PQ) poisoning is one of the greatest clinically important herbicides causing morbidity and mortality. Respiratory failure resulting from lung injury is the most common cause of death from PQ (Dinis-Oliveira et al., 2008). Liu et al. (2014) found that JWH133 mitigated PQ-induced lung edema and pathology. JWH133 also inhibited the release of inflammatory cytokines (TNF-α and IL-1β) in bronchoalveolar lavage fluid, PaO_2_ in arterial blood, and myloperoxidase levels in the lung tissue. This was associated with a remarkable inhibition of phosphorylation of p38MAPK, ERK1/2, JNK1/2, and MAPK and stimulation of NF-kB. CB2R activation in lung tissue protected against PQ-induced acute lung injury by suppressing the stimulation of MAPKs and NF-kB signaling.

#### Asthma

Asthma is a chronic inflammatory airway disease linked with bronchospasm and airway hyperresponsiveness (Groot Kormelink et al., 2009). Frei et al. (2016) found that JWH133 stimulated a moderate migratory response in mice eosinophils. However, short-term treatment with JWH133 augmented chemoattractant-mediated eosinophil shape changes and upregulated adhesion molecules such as CD11b and ICAM-1 as well as increased the release of ROS. However, the effects of JWH133 were abrogated in CB2 knockout mice and after treatment with SR144528. Systemic treatment with JWH133 intensified the eotaxin-2/CCL24-induced eosinophil recruitment into the airways of IL-5Tg mice and aggravated ovalbumin-induced asthma by enhancing eosinophil migration into the lungs and deteriorating airway hyperreactivity in a CB2-dependent manner. This effect was completely reversed in eosinophil-deficient ΔdblGATA mice, indicating that eosinophils could be the main target of JWH133 in allergic inflammation. This CB2-induced triggering of eosinophil influx could be independent of Gi/o/adenylyl cyclase but includes the Gaq/MEK/ROCK pathway. Thus, the cannabinoid/CB2 axis might influence allergic inflammation and indicate possible unwanted inflammatory effects of continuing cannabinoid use.

Similarly, a study by Yeh et al. (2016) found that perivagal treatment with JWH133 did not attenuate H_2_O_2_-induced vagal lung C-fiber hypersensitivity in rats, reflecting the pathophysiology of airway hyperresponsiveness in asthmatic patients (Kuo and Lai, 2008), suggesting that CB2R activation does not reduce the hypersensitivity in vagal lung C-fibers. Furthermore, Bozkurt et al. (2016) reported that JWH133 did not modify serotonin-induced hyperreactivity in tracheas obtained from dinitrofluorobenzene (DNFB) group of non-atopic asthmatic mice. Further, JWH133 did not inhibit the increase in macrophage number in bronchoalveolar lavage fluid. Therefore, CB2R stimulation did not mitigate airway inflammation in DNFB-treated mice.

Gastroesophageal reflux is a prevalent clinical disease linked with several respiratory symptoms, such as bronchoconstriction and chronic cough, and it is more common in patients with asthma (Leggett et al., 2005). Contrary to the nonprotective effect of JWH133 in airway inflammation, a study by Cui et al. (2007) found that JWH133 suppressed microvascular airway leakage and bronchoconstriction induced by intraoesophageal HCl in guinea pigs. However, the protective effect of JWH133 was reversed by SR144528, indicating that the effect was mediated by CB2R stimulation. This finding was consistent with a study conducted by Yoshihara et al. (2004), who found that JWH133 resulted in a dose-dependent inhibition of electrical field stimulation and capsaicin-induced contraction of bronchi obtained from guinea pigs.

Further, JWH133 prevented capsaicin-mediated production of substance P-like immunoreactivity from guinea pig airway tissues, which indicated that CB2R decreased the stimulation of capsaicin-sensitive afferent sensory nerves (C-fibers) in airways. Moreover, Patel et al. (2003) showed that JWH133 administration suppressed cough reflex induced by citric acid in guinea pigs. They also found that JWH133 repressed sensory nerve depolarization of the guinea pig and human vagus nerve induced by hypertonic saline, capsaicin, or the prostaglandin PGE2, whereas this effect was blocked by treatment with SR 144528. Furthermore, Grassin-Delyle et al. (2014) found that the highest concentrations of JWH133 resulted in the suppression of electrical field stimulation-induced contraction of human bronchi with a longer time to onset of action of 167 min.

#### Lung Fibrosis

Pulmonary fibrosis is a group of lung diseases that comprises a combination of inflammation and fibrosis of the lung parenchyma (Gutsche et al., 2012). Fu et al. (2017) reported that preincubation of TGF-β1-activated lung fibroblasts with JWH133 counteracted the induction of collagen I and α-SMA and suppressed fibroblast growth and migration, all of which were reversed by coincubation with SR144528. Preventive dosing with JWH133 reduced lung fibrosis in bleomycin-treated mice and was associated with significant inhibition of the inflammation and extracellular collagen accumulation and reduced hydroxyproline content.

Notably, JWH133 decreased the serum levels of TGF-β1 and repressed TGF-β1/Smad2 signaling *in vitro* and *in vivo*. These data suggest that activation of the CB2R by a pharmacological agent is a promising strategy for pulmonary fibrosis. Wawryk-Gawda et al. (2018) found that JWH133-treated mice showed normal lung tissue structure and thinner alveolar septum compared with nicotine mice. JWH133-treated mice also showed septum thickness and collagen accumulation. JWH133 downregulated the expression of connective tissue growth factor, an essential inducer of pulmonary fibrosis, and α-SMA, suggesting its beneficial function in preventing interstitial fibrosis.

#### Lung Ischemia-Reperfusion Injury

Lung ischemia–reperfusion injury (IRI) is a common and severe postoperative complication after cardiopulmonary bypass, lung transplantation, pulmonary thrombosis, and cardiac arrest (den Hengst et al., 2010). Zeng et al. (2019) reported that pretreatment with JWH133 mitigated lung edema and infiltration of inflammatory cells and lung histopathological alternations induced by IRI in mice. Further, JWH133 administration ameliorated the PaO2/FiO2 ratio, reduced lung TNF-α, IL-6, MDA levels, myeloperoxidase activities, and enhanced superoxide dismutase activity. However, the beneficial effects of JWH133 were abrogated by pretreatment with AM630, indicating that CB2R stimulation prevented IR-mediated oxidative injury and inflammatory response and improved lung IRI.

Likewise, pretreatment with a PI3K inhibitor reversed the protective effect of JWH133 and decreased the expression of *p*-AKT without altering JWH133-driven CB2R expression. Thus, CB2R activation could protect against IR-mediated lung injury by attenuating inflammation and oxidative stress in mice via PI3K/AKT signaling. Huang et al. (2020) showed that pretreatment with JWH133 markedly mitigated the lung injury induced by I/R and reduced oxidative stress in mice. It also led to a significant upregulation of expression of CB2R and downregulation of NOX2. In contrast, cotreatment with AM630 or a NOX2 inhibitor reversed the effects of JWH133. Therefore, CB2R stimulation alleviated lung IRI by inhibiting oxidative stress via NOX2 in mice.

### JWH133 in Viral Infections

Respiratory syncytial virus (RSV) causes severe lower respiratory tract symptoms, mainly bronchiolitis and pneumonia, in infants and young children (Borchers et al., 2013; Troy and Bosco, 2016). Tahamtan et al. (2018) reported an association between CB2 variant Q63R and a high risk of hospitalization in children with acute respiratory tract infection. Further, children with the QQ genotype were more vulnerable to severe acute respiratory tract infection. The increased risk of developing severe acute respiratory tract infection secondary to RSV infection is more than 2-fold higher in children who carry the Q allele. In Balb/c mice, JWH133 significantly reduced the influx of bronchoalveolar lavage (BAL) cells and abolished leukocyte migration into the lungs in RSV.

Moreover, CB2 stimulation by JWH133 resulted in a significant reduction in the levels of IFN-γ and MIP-1α and increased IL-10 levels in the BAL of mice while mitigating lung pathology. JWH133 also inhibited the accumulation of immune cells in the peribronchial and perivascular spaces of the lung after RSV infection. Therefore, CB2R is the primary signaling pathway for endocannabinoid-mediated immune modulation and might play a pivotal role in regulating immune homeostasis and maintaining the extent of the immunological response via a negative regulatory mechanism.

### JWH133 in Wound Repair, Healing, and Differentiation

Several studies have demonstrated that cannabinoids promote wound healing by enhancing cellular migration, resulting in the preservation of vascular integrity (Zhang et al., 2010); corneal wound healing (Yang et al., 2010); and epithelial wound closure in colonic tissues (Wright et al., 2005). CB2Rs have demonstrated wound healing effects in various models (Li et al., 2016; Wang et al., 2016). Ruhl et al. (2020) found that incubation with JWH133 elevated the population of human adipose tissue mesenchymal stromal cells (atMSC), which release many cytokines and growth factors that control cell differentiation, angiogenesis, and the immune response to mediate the repair of damaged tissue (Yancopoulos et al., 2000; Pakyari et al., 2013). JWH133 enhanced VEGF, TGF-β1, and HGF secretion, which then enhanced the regenerative activity of atMSCs. Thus, CB2R agonists could be a promising target for increasing the regenerative potential of atMSCs.

Schmuhl et al. (2014) showed that JWH133 stimulated the migration of mesenchymal stem cells, which mediate wound healing. This effect was suppressed by AM-630 and the p42/44 MAPK activation antagonist PD98059, indicating that CB2R stimulation by JWH133 induces p42/44 MAPK. Furthermore, JWH133 mitigated TGF-β1-mediated production of fibronectin, collagen I and III, and expression of MMP-1 and MMP-3 in cultured human Tenon’s fibroblasts (Guan et al., 2017). JWH133 also attenuated TGF-β1-mediated matrix contraction and remodeling in a dose-dependent manner, in conjunction with a remarkable suppression of activated MAPKs, such as ERK1/2, p38, and JNK as well as extracellular matrix synthesis and the contractility of human Tenon’s fibroblasts *in vitro*. Therefore, pharmacological stimulation of CB2R could protect against scar formation in wound healing after glaucoma filtration surgery. Murataeva et al. (2019) showed that CB2R activation by JWH133 induced a chemorepulsive effect in cultured corneal epithelial cells (CECs), but did not change CEC growth. CB2R activation also induced *p*-ERK expression and cAMP production, the latter being due to Gs-coupling. Additionally, wound closure was delayed in CB2R-knockout mice and the presence of CB2R blockade by SR144528. Thus, CB2R receptor activation could support wound healing, possibly by chemorepulsion.

The physiological balance between self-renewal and differentiation is necessary for hematopoietic stem cell function and hematopoiesis. CB2Rs localize to human and murine hematopoietic stem and progenitor cells (HSPCs), and JWH133 activation induces colony formation and HSPC recruitment *in vitro* and accelerates colony formation of bone marrow cells via ERK, PI3-kinase, and Gαi-Rac1 signaling (Jiang et al. 2011). However, granulocyte colony-stimulating factor-stimulated migration of HSPCs was significantly attenuated by AM630 and was absent in CB2^−/−^ mice. These findings implicate the cannabinoid system in hematopoiesis and suggest that CB2 activation mediates repopulation and migration of HSPCs, indicating its clinical value in bone marrow transplantation.

JWH133 augmented oligodendrocyte progenitor cell differentiation, as demonstrated by the increased expression of stage-specific antigens and myelin basic protein, and this effect was reversed by AM630 (Gomez et al., 2011). Enhanced oligodendrocyte differentiation was owing to the JWH133-stimulated CB2R activation of *p*-AKT and mTOR signaling. Therefore, CB2R stimulation could profoundly affect oligodendrocytes and consequently affect brain repair. In a rat model of skeletal muscle contusion, CB2R activation by JWH133 significantly reduced the fibrotic area and inhibited the expression of collagen type I/ІІІ as well as amplified the number of multinucleated regenerating myofibers in the injured area (Yu et al., 2015). These results were directly attributed to the reduced expression of TGF-β1, fibronectin-EIIIA, and α-SMA; decreased production of myofibroblasts; and concurrent upregulation of MMP-1/2 by JWH133. Therefore, CB2R activation inhibited fibrotic formation and improved muscle regeneration, suggesting a therapeutic value in patients with skeletal muscle injuries and disorders. JWH133 stimulation of CB2R attenuated the infiltration of M1 macrophages and enhanced M2 populations in a mouse model of incised skin wound healing (Du et al. 2018). JWH133 also downregulated the expression of the M1-related cytokines IL-6, IL-12, CD86, and iNOS and upregulated the expression of the M2-related cytokines IL-4, IL-10, CD206, and Arg-1. Inhibition of the inflammatory process by CBR2 activation might lead to the development of novel therapies for cutaneous inflammation.

## Conclusion

JWH133 is a synthetic cannabinoid with seemingly limitless therapeutic potential for different pathological conditions, primarily owing to its CB2R specificity, which in addition to making this synthetic ligand devoid of psychoactive effects, determines its major biological activities. The available studies reviewed here suggest that JWH133 inhibits inflammation, oxidative stress, and apoptosis, among other effects, resulting in the mitigation of various pathologies. JWH133 is considered a suitable CB2R agonist for preclinical target validation, based on the following features: 1) selective agonism on CB2R over CB1R in both humans and mice, 2) well-balanced stimulation of signaling transduction on human CB2R, 3) negligeable number of off-target activities at its effective doses, 4) reasonable pharmacokinetics properities and 5) deficiency of cannabimimetic pharmacological effects *in vivo* suggestive of CB1R activity. The bulk of our knowledge about the polypharmacological effects of JWH133 in *in vitro* and *in vivo* models is derived from the aforementioned studies. Much of this work displayed potent antioxidant, anti-inflammatory, and antiapoptotic activities, confirmed *in vitro* and *in vivo* mechanisms of JWH133 actions and could allow a successful transfer of preclinical data to the patient’s bedside. However, further investigations in animals are needed to delineate the pharmacokinetic properties as well as safety and toxicity of JWH133 before large scale human studies are conducted. Such investigations may recognize more clinically suitable routes of administration, establish the extent of drug stability and metabolism while providing evidences about potential adverse effects of JWH133. As the U.S. Drug Enforcement Administration criminalizes any extract “containing one or more cannabinoids,” JWH133 is a scheduled substance in the U.S. This is despite the low addictive potential relative to its sister compounds such as JWH-018, as JWH133 is highly selective for the non-psychoactive CB2R and thus lacks significant psychoactive effects.
